# Predicting response to immunochemotherapy in EGFR-mutant lung adenocarcinoma after third-generation TKI resistance using CT radiomics-based habitat imaging

**DOI:** 10.3389/fimmu.2026.1785968

**Published:** 2026-06-02

**Authors:** Shuai Qie, Yasong Shi, Jingyun Li, Sicong Jia, Xiaoping Yin

**Affiliations:** Department of Radiation Oncology, Affiliated Hospital of Hebei University, Baoding, Hebei, China

**Keywords:** EGFR mutation, habitat imaging, immunochemotherapy, lung adenocarcinoma, radiomics

## Abstract

**Background:**

Third-generation epidermal growth factor receptor (EGFR)-tyrosine kinase inhibitor (TKI) resistance poses a significant therapeutic challenge in advanced lung adenocarcinoma. This study aimed to develop and validate a computed tomography (CT)-based habitat radiomics model for predicting response to immunochemotherapy in EGFR-mutant lung adenocarcinoma patients after TKI resistance.

**Methods:**

This retrospective multicenter study enrolled 475 patients from two medical centers. Patients were allocated to train (N = 332) and external validation (N = 143) cohorts. Habitat imaging was performed using K-means clustering to partition tumors into three distinct subregions. Radiomic features were extracted from both whole-tumor volumes and habitat subregions. A combined model combining clinical, conventional radiomics, and habitat features was constructed using machine learning algorithms and validated through cross-validation and external testing. The primary endpoint was objective response rate (ORR) based on Response Evaluation Criteria in Solid Tumors (RECIST) 1.1 criteria, and overall survival (OS) was used as a secondary endpoint.

**Results:**

The combined model demonstrated superior predictive performance with area under the curve (AUC) of 0.904 (95% CI: 0.871–0.937) in the train cohort and 0.890 (95% CI: 0.838–0.942) in the validation cohort, significantly outperforming the clinical model, conventional whole-tumor radiomics model, and habitat model (all P < 0.001). Moreover, Kaplan–Meier analysis based on the risk groups stratified by the combined model revealed significant survival differences, with high-risk groups showing markedly shorter overall survival in both cohorts (training HR = 3.688, validation HR = 2.823, both log-rank P < 0.0001).

**Conclusion:**

This study developed and externally validated a CT-based habitat radiomics model for predicting response to immunochemotherapy in EGFR-mutant lung adenocarcinoma after EGFR-TKI resistance. The combined model achieved improved predictive performance compared with single-modality approaches. These findings suggest that incorporating habitat-based features may enhance the characterization of intratumoral heterogeneity and improve treatment response prediction. Notably, the model demonstrated a high negative predictive value, suggesting its potential to reduce unnecessary treatment in predicted non-responders. Further prospective and multi-center validation is warranted.

## Introduction

Lung adenocarcinoma driven by EGFR mutations is commonly treated with third-generation TKIs, yet acquired resistance remains a major clinical challenge (1–3). Following resistance, treatment options are limited, with immune checkpoint inhibitors combined with platinum-based chemotherapy (immunochemotherapy) being a potential strategy (4). Nonetheless, the efficacy of this regimen varies significantly among patients. Consequently, accurately identifying which patients with EGFR-mutant lung adenocarcinoma are likely to benefit from immunochemotherapy after third-generation TKI failure represents a critical, unmet need in current precision oncology.

Current clinical decision-making primarily relies on limited clinicopathological features, lacking robust predictive biomarkers (5, 6). Several biomarkers have been investigated to predict response to immunochemotherapy in EGFR-mutant lung adenocarcinoma following TKI resistance. Molecular alterations associated with resistance, such as secondary EGFR mutations and bypass pathway activation (e.g., MET amplification), have been reported to influence treatment response. In addition, tumor mutational burden (TMB) and tumor-infiltrating lymphocytes (TILs) have been explored as potential indicators of immunotherapy efficacy. However, these biomarkers have several important limitations. First, most are based on invasive tissue sampling, which may not fully capture tumor heterogeneity. Second, both spatial and temporal heterogeneity of tumors may lead to sampling bias and inconsistent results. Third, the predictive performance of these biomarkers in EGFR-mutant populations remains suboptimal and inconsistent across studies. Therefore, there is a critical need for non-invasive and reproducible biomarkers that can better characterize intratumoral heterogeneity and improve treatment stratification.

Conventional medical imaging assessments, such as RECIST criteria (7), only provide macroscopic information on tumor size changes and cannot predict treatment response prior to therapy initiation. Although radiomics, a technique that extracts high-throughput quantitative features from medical images, has shown promise in cancer diagnosis and prognosis prediction, most studies still analyze tumors as homogeneous entities. This approach potentially overlooks the profound intra-tumoral spatial heterogeneity—a key biological characteristic associated with malignant progression and treatment resistance. Therefore, developing novel imaging biomarkers capable of characterizing intratumoral heterogeneity is of paramount importance for addressing this clinical dilemma.

Habitat imaging has also been increasingly applied in lung cancer to characterize intratumoral heterogeneity and improve prediction of treatment response and prognosis (8–10). Previous studies have demonstrated that habitat-based radiomics can capture spatially distinct tumor subregions associated with biological heterogeneity and clinical outcomes (11, 12). However, most existing studies have focused on general non-small cell lung cancer populations or conventional treatment settings. The application of habitat imaging in EGFR-mutant lung adenocarcinoma, particularly after targeted therapy resistance and in the context of immunochemotherapy, remains limited. Therefore, its value in predicting response to immunochemotherapy in EGFR-mutant lung adenocarcinoma following TKI resistance remains insufficiently explored. Accordingly, this study aimed to develop and validate a CT-based habitat radiomics model to predict treatment response (ORR) prior to therapy initiation, and to further evaluate its prognostic value using OS.

## Methods

### Patients and data

This was a retrospective, multicenter study conducted in accordance with the Declaration of Helsinki. The protocol was approved by the institutional review boards of all participating centers (Affiliated Hospital of Hebei University, approval No. HDFY-LL-2022–075; Baoding First Central Hospital, approval no. BFCH-LL-2023–016). The requirement for informed consent was waived due to the retrospective nature of the study and the use of anonymized data.

**Figure 1:**
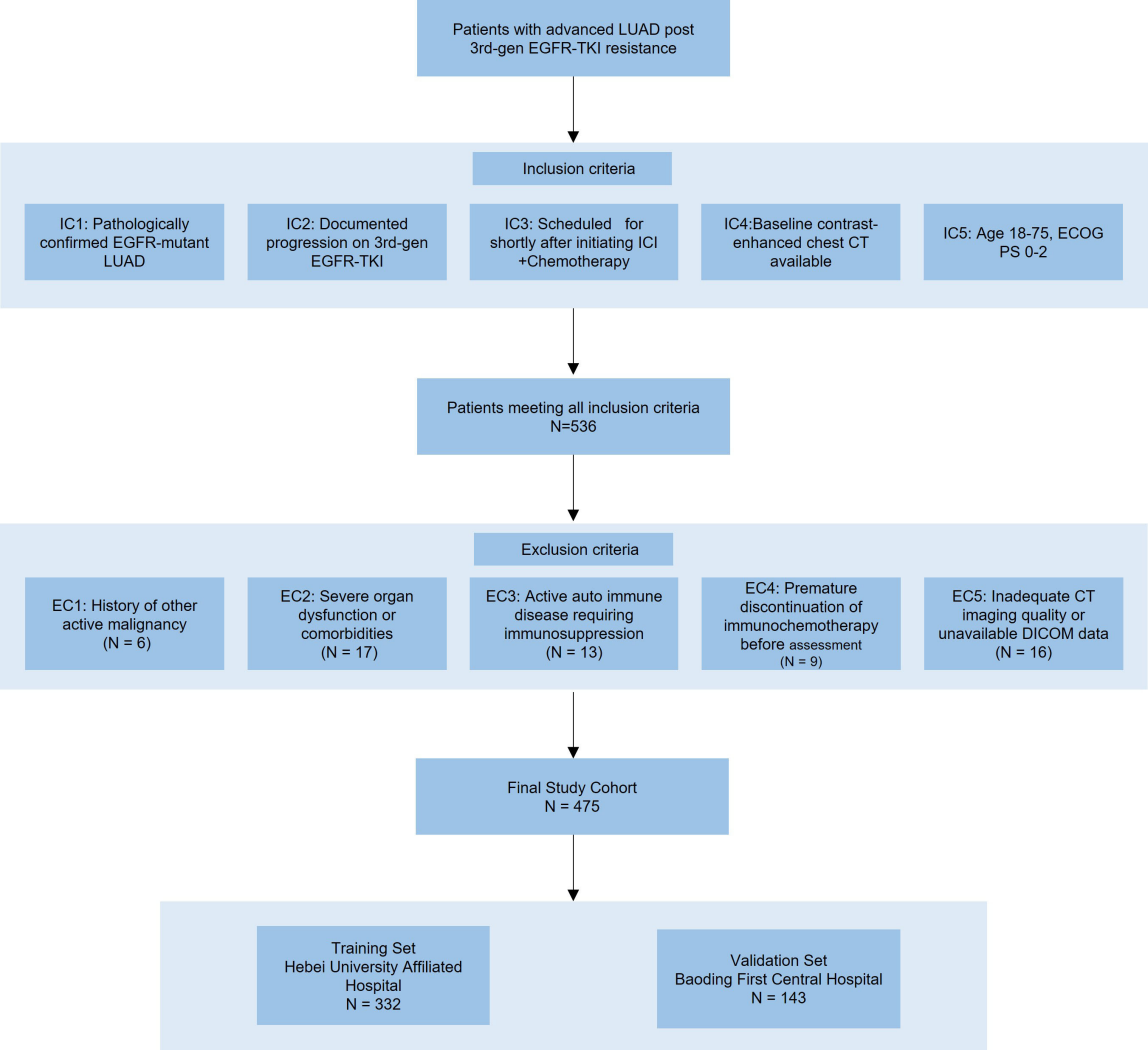
Flowchart shows patient exclusion for each dataset.

We screened consecutive patients with advanced lung adenocarcinoma treated at the two centers between January 2018 and December 2023 at both centers. Inclusion criteria were: 1) histopathologically confirmed stage IV or recurrent lung adenocarcinoma; 2) EGFR-sensitive mutations (exon 19 deletion or L858R substitution); 3) confirmed clinical and radiological progression (per RECIST 1.1) after first-line EGFR-TKI treatment, defining acquired resistance; 4) first subsequent treatment with immune checkpoint inhibitors plus platinum-based chemotherapy; 5) availability of baseline contrast-enhanced chest CT within 4 weeks before immunochemotherapy; and 6) complete clinical and follow-up data. Exclusion criteria were: 1) prior immunotherapy; 2) poor-quality baseline CT preventing accurate segmentation/analysis; 3) <2 cycles of immunochemotherapy; 4) concurrent active malignancy; and 5) severely incomplete clinical records. The patient selection process is shown in **Figure 1**.

A total of 475 eligible patients were enrolled. For model development and validation, patients from the Affiliated Hospital of Hebei University (N = 332) constituted the training cohort, while patients from Baoding First Central Hospital (N = 143) served as a fully independent external validation cohort. The validation cohort consisted entirely of patients from an independent center, ensuring strict external validation with no overlap between cohorts. All eligible patients from the external center were included to minimize selection bias. No data from the external validation cohort were used for model training or hyperparameter tuning. Baseline characteristics are summarized in **Table 1**. No significant differences were observed between the training and validation cohorts (all P > 0.05), confirming their comparability.

**Table 1:** Clinical characteristics of patients in training and validation cohorts.

Feature name	ALL	Train	Validation	P value
Age	61.81±10.10	61.79±10.37	61.85±9.48	0.903
Sex				0.561
Female	308 (64.84)	212 (63.86)	96 (67.13)	
Male	167 (35.16)	120 (36.14)	47 (32.87)	
T stage				0.222
T1	175 (36.84)	118 (35.54)	57 (39.86)	
T2	116 (24.42)	77 (23.19)	39 (27.27)	
T3	22 (4.63)	14 (4.22)	8 (5.59)	
T4	162 (34.11)	123 (37.05)	39 (27.27)	
N stage				0.285
N0	92 (19.37)	57 (17.17)	35 (24.48)	
N1	141 (29.68)	102 (30.72)	39 (27.27)	
N2	109 (22.95)	80 (24.10)	29 (20.28)	
N3	133 (28.00)	93 (28.01)	40 (27.97)	
Smoking status				0.502
No	354 (74.53)	244 (73.49)	110 (76.92)	
Yes	121 (25.47)	88 (26.51)	33 (23.08)	
Bone metastases				0.952
No	293 (61.68)	204 (61.45)	89 (62.24)	
Yes	182 (38.32)	128 (38.55)	54 (37.76)	
Liver metastases				0.987
No	427 (89.89)	299 (90.06)	128 (89.51)	
Yes	48 (10.11)	33 (9.94)	15 (10.49)	
Pleural effusion				0.565
No	313 (65.89)	222 (66.87)	91 (63.64)	
Yes	162 (34.11)	110 (33.13)	52 (36.36)	
Pulmonary metastasis				0.887
No	325 (68.42)	226 (68.07)	99 (69.23)	
Yes	150 (31.58)	106 (31.93)	44 (30.77)	
Distant lymph node metastasis				0.551
No	388 (81.68)	274 (82.53)	114 (79.72)	
Yes	87 (18.32)	58 (17.47)	29 (20.28)	
EGFR mutations				0.542
19del	192 (40.42)	138 (41.57)	54 (37.76)	
21 L858R	188 (39.58)	126 (37.95)	62 (43.36)	
Other	95 (20.00)	68 (20.48)	27 (18.88)	
Primary site				0.417
Left	201 (42.32)	145 (43.67)	56 (39.16)	
Right	274 (57.68)	187 (56.33)	87 (60.84)	

### Treatment regimen and response assessment

**Figure 2:**
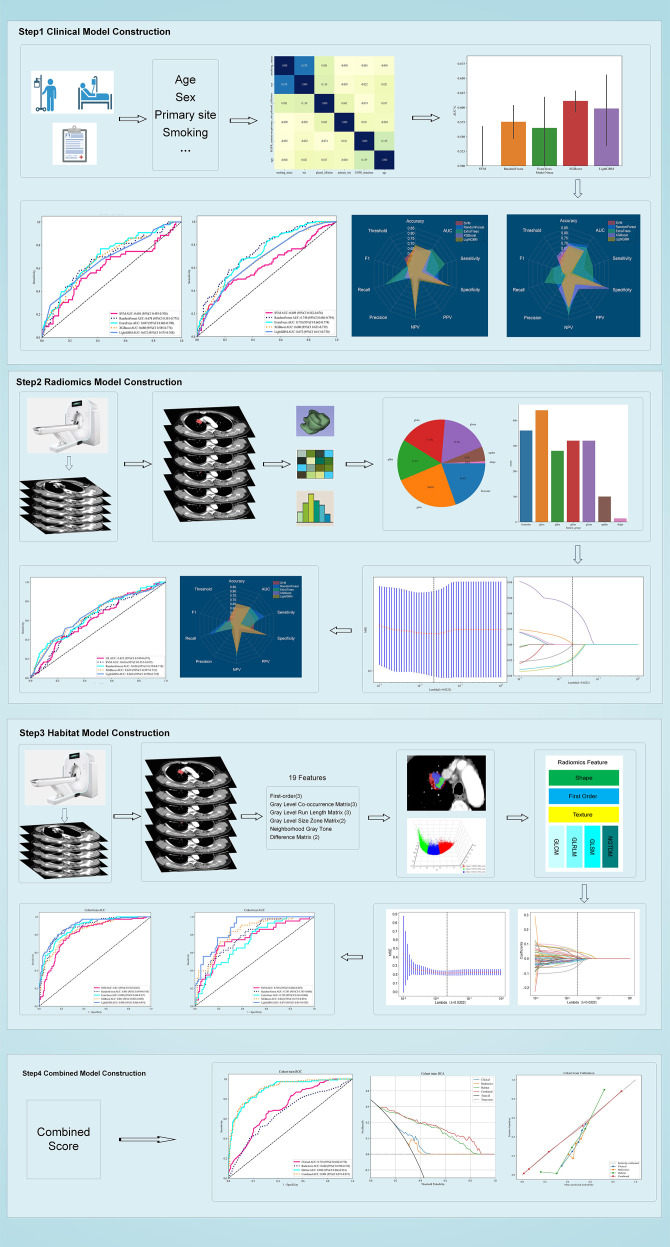
The study’s flowchart.

All patients received standard third-generation EGFR-TKIs until radiological progression per RECIST 1.1. They then started first-line immunochemotherapy. To standardize treatment for analysis, the regimen was defined as a PD-1 inhibitor (pembrolizumab 200 mg or Sintilimab 200 mg, IV on day 1) plus platinum-based chemotherapy (pemetrexed 500 mg/m² and carboplatin AUC = 5, IV on day 1) in 21-day cycles. Treatment continued until disease progression, unacceptable toxicity, or patient withdrawal. Treatment response was the primary endpoint. Two independent radiologists (with 8 and 15 years of thoracic imaging experience), blinded to clinical and modeling data, assessed response. Patients underwent chest/abdominal CT at baseline and every 6–8 weeks (~2–3 cycles) after starting immunochemotherapy. Response was categorized per RECIST 1.1 as complete response (CR), partial response (PR), stable disease (SD), or progressive disease (PD). For binary classification in modeling, patients achieving CR or PR were classified as responders, while those with SD or PD were classified as non-responders. Adverse events were recorded and graded using CTCAE v5.0. The overall workflow is shown in **Figure 2**.

### Endpoints and definitions

The primary endpoint was ORR evaluated according to RECIST 1.1 criteria. OS was defined as the time from treatment initiation to death from any cause and was used for survival analysis.

### CT image acquisition and tumor segmentation

CT acquisition parameters are detailed in **Supplementary Table 1**. In brief, arterial-phase contrast-enhanced chest CT was performed after intravenous administration of a non-ionic iodine contrast agent (350 mg I/mL; dose 60–80 mL; rate 2–3 mL/s), covering the lung apex to the costophrenic angles. Images were reconstructed using a standard/high-resolution algorithm and retrieved in DICOM format from PACS. For habitat analysis, the entire tumor volume was defined as the volume of interest (VOI). Two blinded readers independently performed 3D manual segmentation using ITK-SNAP (v3.8.0), with contours encompassing the whole tumor. All segmentations were reviewed and verified by senior specialists, with discrepancies resolved by consensus. To assess reproducibility, a random subset of 50 cases was re-segmented by both readers. Features with an intra-class correlation coefficient (ICC) ≥0.75 in both intra- and inter-observer analyses were retained.

### CT preprocessing

To ensure robustness and comparability of radiomic features across different centers, a standardized image preprocessing pipeline was applied. First, all CT images were resampled to isotropic voxel spacing (1×1×1 mm³) to eliminate differences in spatial resolution. Intensity normalization was then performed to reduce variability in gray-level distributions across scans. Subsequently, image discretization was performed using a fixed bin width to ensure reproducibility of radiomic features. CT acquisition parameters, including tube voltage, tube current, slice thickness, and reconstruction algorithms, were collected and are summarized in **Supplementary Table 1**. Although minor variations existed across centers, standardized preprocessing procedures, including voxel resampling and intensity normalization, were applied to reduce inter-scanner variability and improve feature robustness.

### Tumor habitat map generation

**Figure 3:**
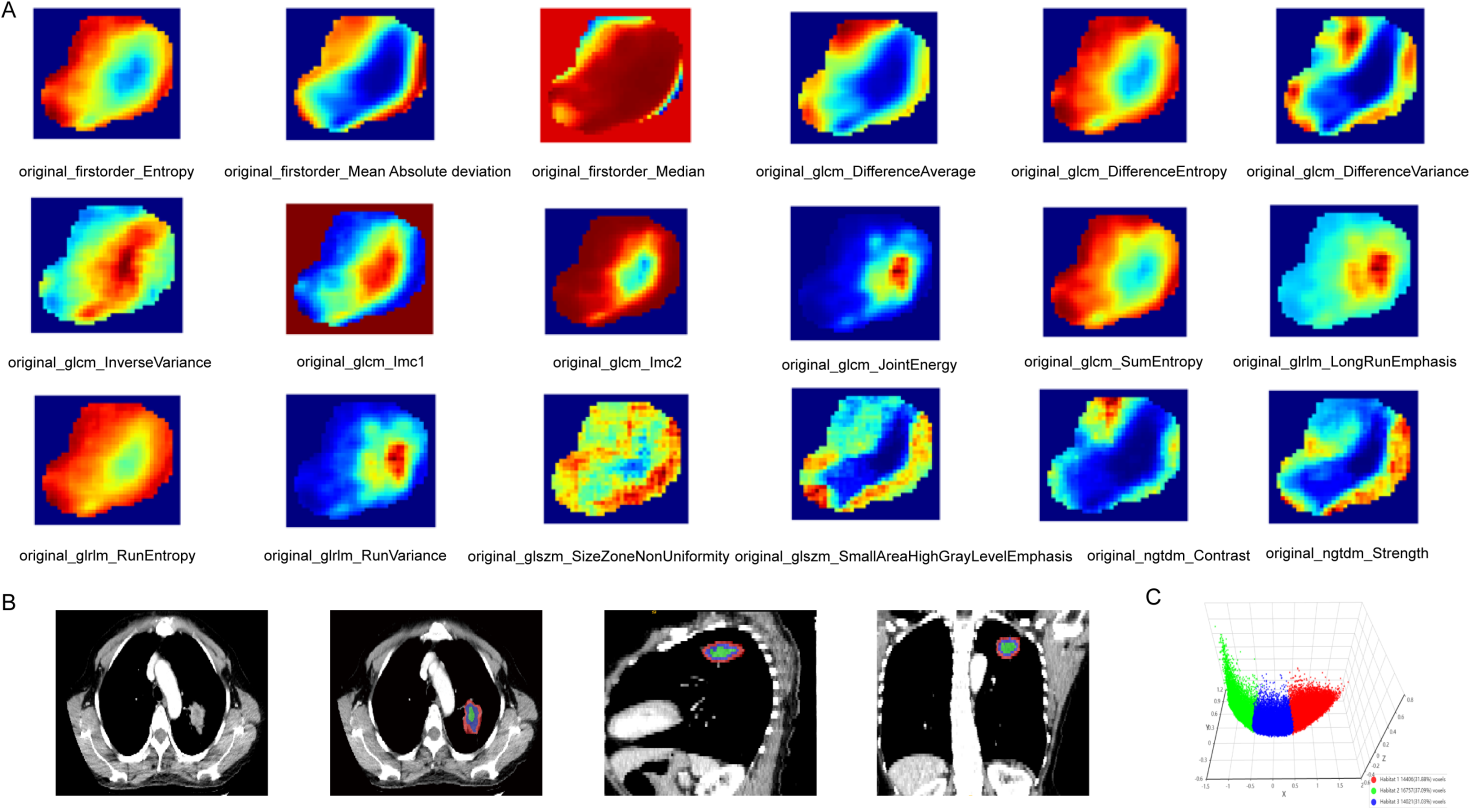
Tumor habitat imaging process. **(A)** Representative radiomic feature maps (e.g., Entropy, GLCM Difference Entropy) derived from CT, color-coded to visualize intra-tumoral phenotypic variation. **(B)** Visualization of cluster numbers in a representative patient. **(C)** Three-dimensional scatter plot illustrating the unsupervised clustering of tumor subregions into three distinct imaging habitats (red, blue, green) within the radiomic feature space.

To construct the tumor habitat map, we first quantified local heterogeneity at the voxel level. To quantify intratumoral heterogeneity, each tumor ROI was partitioned into 100 subregions using the Simple Linear Iterative Clustering super pixel algorithm prior to habitat clustering analysis. (Detailed parameters and algorithm formulation are provided in the **Supplementary S1**). The specific pipeline was as follows: Based on the standardized radiomic feature maps extracted from the whole-tumor VOI of each patient, a 3×3×3 cubic neighborhood operator was applied to compute a set of feature statistics (e.g., entropy, energy) for each voxel within its local context. This process generated a local feature vector comprising 19 descriptors per voxel, thereby representing the entire tumor as a high-dimensional per-voxel feature space (**Figures 3A, B**).

Subsequently, an unsupervised K-means clustering algorithm was applied to this feature space to identify subregions (i.e., habitats) with similar imaging phenotypes. The optimal number of clusters (k) was determined by comprehensively evaluating the Calinski-Harabasz index, Silhouette score, and Davies-Bouldin index (**Supplementary Figure S1**). Internal validation indicated that k = 3 provided the optimal balance across these metrics, which is consistent with the biological rationale of a tripartite model for characterizing intratumoral heterogeneity, as reported in previous radiomics studies. Consequently, the voxels within each tumor were classified into three distinct imaging habitats (**Figure 3C**). The stability of the habitat segmentation was assessed by repeating the entire clustering procedure on the reproducibility subset. Clustering-derived features demonstrated good reproducibility, with ICC values ranging from 0.75 to 0.92, indicating that the habitat segmentation process is robust and reproducible.

## Feature extraction

### Whole-tumor feature extraction

Radiomic features from each subregion were computed with PyRadiomics (version 3.0.1), following the Image Biomarker Standardization Initiative (IBSI) standards (13). Standardized radiomic features (including shape, first-order, and texture features) were extracted from the whole-tumor VOI, as detailed in **Supplementary Table 2**.

### Habitat feature extraction

Radiomic features were extracted from each of the three clustered tumor subregions (habitats) using PyRadiomics based on the original CT image. As the morphological characteristics of individual subregions were not the focus of this analysis, shape features were excluded. Therefore, for each habitat, the extracted radiomic features included first-order statistics (N = 18) and texture features from GLCM (N = 24), GLRLM (N = 16), GLSZM (N = 16), NGTDM (N = 5), and GLDM (N = 14). The total number of features extracted from each habitat sub-region, categorized by type, is detailed in **Supplementary Table 3**. The features extracted from habitat 1, 2, and 3 were concatenated and prefixed as feature_h1, feature_h2, and feature_h3, respectively, to form the comprehensive habitat-based feature vector for modeling.

### Feature selection and predictive model construction

To develop robust predictive models while mitigating the risk of overfitting from high-dimensional data, a rigorous feature selection and preprocessing pipeline was applied separately to the whole-tumor and habitat feature sets. Intraclass correlation coefficients (ICCs) were calculated using a two-way random-effects model to assess both intra- and inter-observer reproducibility, and features with ICC ≥ 0.75 were retained. Subsequently, features were normalized using Z-score transformation, followed by Spearman’s correlation analysis to remove redundant features. Least Absolute Shrinkage and Selection Operator (LASSO) regression was then applied, and the optimal regularization parameter (λ) was determined using 10-fold cross-validation based on the minimum criteria to select the most predictive feature subset. The detailed feature selection process and intermediate results are provided in the **Supplementary Material**. Feature selection results and corresponding coefficients are provided in **Supplementary Table 4**.

### Model evaluation and statistical analysis

Performance was assessed on the independent validation cohort. The primary metric was the AUC, compared pairwise via DeLong test (P < 0.05). Secondary metrics (accuracy, sensitivity, specificity, PPV, NPV) were derived from the Youden index-optimized threshold. Calibration (calibration curves, Hosmer-Lemeshow test) and clinical utility (decision curve analysis, DCA) of the combined model were also evaluated. Categorical variables are presented as frequencies/percentages and compared using Chi-square or Fisher’s exact test. Continuous variables, expressed as mean ± SD or median with IQR, were compared using t-tests or Mann-Whitney U tests, as appropriate. Multivariate Cox regression analysis was performed adjusting for age, sex, TNM stage, and EGFR mutation subtype. All analyses were performed in R (v4.3.1).

## Results

### Patient characteristics

A total of 475 patients with advanced lung adenocarcinoma were included, comprising a training cohort (N = 332) and an independent validation cohort (N = 143). The overall population had a mean age of 61.81 ± 10.10 years and was predominantly female (64.84%) and never-smokers (74.53%). The most common primary tumor stages were T1 (36.84%) and T4 (34.11%), while N2 represented the most frequent nodal stage (22.95%). Bone metastasis was the most prevalent metastatic site (38.32%), followed by pulmonary metastasis (31.58%). EGFR mutation subtypes were mainly exon 19 deletion (40.42%) and exon 21 L858R (39.58%). Importantly, no significant differences were observed between the training and validation cohorts across all baseline variables (all P > 0.05), indicating good comparability (**Table 1**). Baseline characteristics stratified by treatment response in both the training and validation cohorts are presented in **Supplementary Table 5** and **Supplementary Table 6**. In the training cohort, T stage, N stage, bone metastases, and EGFR mutation subtype showed statistically significant differences between responders and non-responders. In the validation cohort, age, T stage, N stage, pulmonary metastasis, and primary tumor site were significantly associated with treatment response. These findings indicate that clinical variables associated with treatment response were not entirely consistent across cohorts, which may reflect differences in patient composition and sample size. This further highlights the need for robust predictive models that are not solely dependent on specific clinical variables.

### Performance of the clinical model

Multivariate analysis identified EGFR mutation subtype as the only independent predictor of treatment response, with exon 19 deletion associated with a lower probability of response (OR = 0.725, 95% CI: 0.559–0.942, P < 0.05) (**Supplementary Table 7**). The clinical model demonstrated limited discriminative ability, achieving an AUC of 0.740 (95% CI: 0.686–0.794) in the training cohort and 0.678 (95% CI: 0.581–0.775) in the validation cohort (**Supplementary Figure S2**; **Supplementary Table 8**). These findings suggest that conventional clinical variables alone are insufficient for accurately predicting response to immunochemotherapy.

### Performance of the radiomics model

The conventional radiomics model showed modest predictive performance. On the validation cohort, it achieved an AUC of 0.657 (95% CI: 0.605–0.751), with an accuracy of 0.678, sensitivity of 0.605, and specificity of 0.710 (**Supplementary Figure S3**; **Supplementary Table 9**). Compared with the clinical model, the radiomics model did not demonstrate substantial improvement, indicating that whole-tumor radiomic features may have limited ability to capture the complex biological heterogeneity associated with treatment response.

### Performance of the habitat model

**Figure 4:**
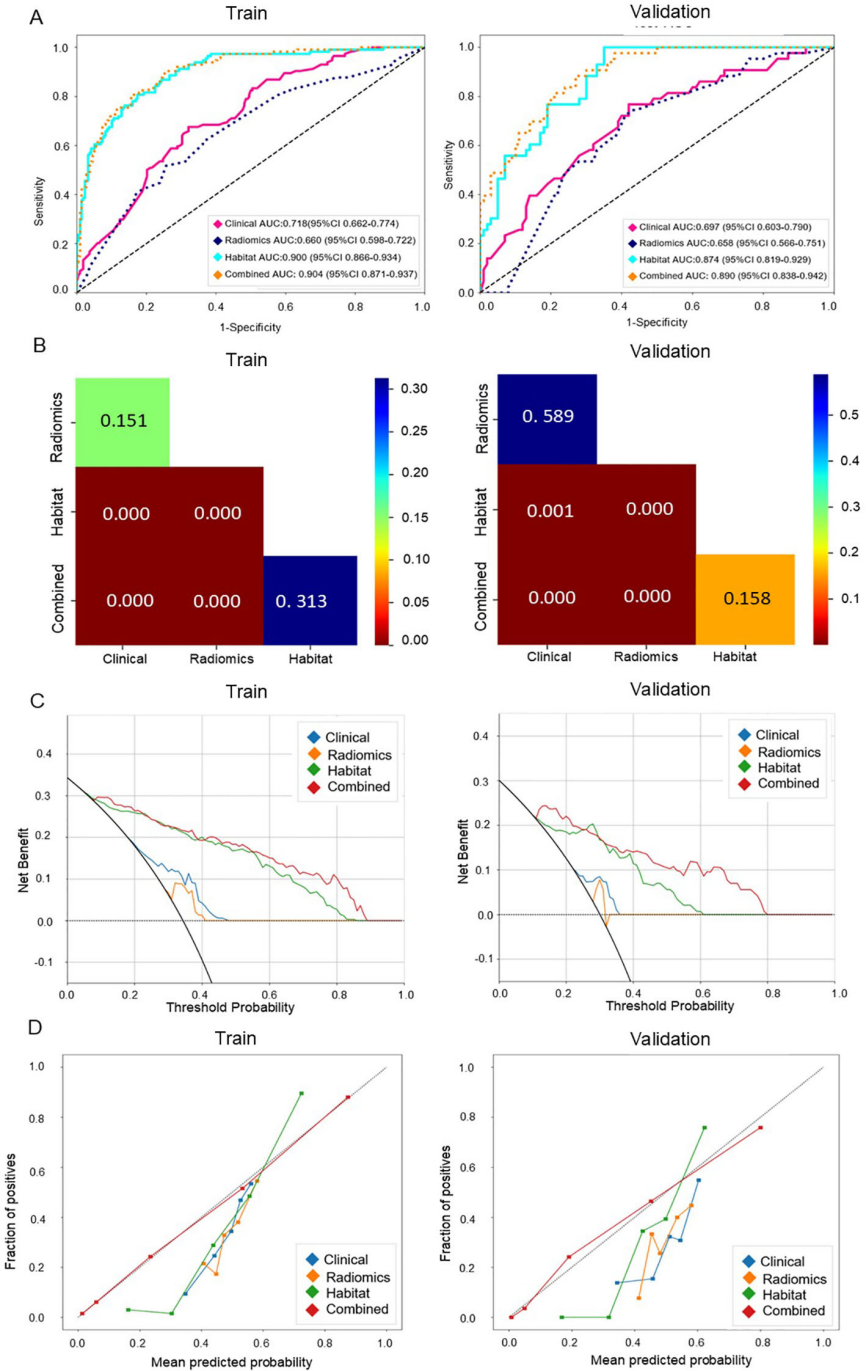
Performance comparison of different models in the training and validation cohorts. **(A)** Receiver operating characteristic (ROC) curves of the clinical, radiomics, habitat, and combined models. **(B)** Pairwise comparison of model performance using the DeLong test. **(C)** Decision curve analysis (DCA) showing the net clinical benefit across different threshold probabilities. **(D)** Calibration curves demonstrating agreement between predicted and observed outcomes.

The habitat model exhibited strong predictive performance, achieving an AUC of 0.900 (95% CI: 0.866–0.934) in the training cohort and 0.874 (95% CI: 0.819–0.929) in the validation cohort (**Figure 4A**; **Supplementary Table 10**). On the validation cohort, the model achieved an accuracy of 0.748, sensitivity of 0.977, and specificity of 0.650. No additional threshold adjustment was performed beyond the Youden index in the current analysis. Notably, the model achieved an NPV of 93.1% in the training cohort and 92.4% in the validation cohort, indicating a strong ability to correctly identify non-responders. The habitat model demonstrated superior performance compared with the conventional radiomics model. To further evaluate whether this improvement was attributable to feature representation rather than algorithm selection, additional analyses were conducted using the same machine learning algorithm (LightGBM) for both models. The results showed that the habitat-based model consistently outperformed the radiomics model in both the training cohort (AUC: 0.900 vs. 0.660) and the validation cohort (AUC: 0.874 vs. 0.658) (**Supplementary Figure S4**).

To improve interpretability of the habitat model, the two most contributory features were further analyzed. As shown in**Supplementary Figure 5**, wavelet_LHH_glszm_SmallAreaEmphasis_h3 was significantly higher in responders than in non-responders, whereas wavelet_LLL_glszm_ZoneVariance_h3 showed higher values in non-responders (P < 0.001). Although the overall distribution shapes of these features were similar between groups, a clear shift in feature values was observed, indicating significant differences between responders and non-responders.

### Performance of the combined model

The combined model demonstrated the best overall performance among all models. It achieved an AUC of 0.904 (95% CI: 0.871–0.937) in the training cohort and 0.890 (95% CI: 0.838–0.942) in the validation cohort (**Figure 4A**; **Supplementary Table 11**). Pairwise comparisons using the DeLong test demonstrated that the combined model significantly outperformed the clinical model (AUC difference = 0.287, 95% CI: 0.167–0.408, Z = 4.64, P < 0.001) and the conventional radiomics model (AUC difference = 0.276, 95% CI: 0.181–0.375, Z = 5.61, P < 0.001). No significant difference was observed between the combined model and the habitat model (AUC difference = 0.017, 95% CI: −0.007–0.041, Z = 1.38, P = 0.158). Detailed pairwise comparison results are provided in **Figure 4B** and **Supplementary Table 12**. Calibration analysis showed good agreement between predicted and observed outcomes, while decision curve analysis demonstrated that the combined model provided the greatest net clinical benefit across threshold probabilities of 10%–80% (**Figures 4C, D**).

Calibration curves of all models are presented in **Supplementary Figures S6A, B**, showing that the combined model achieved the best agreement between predicted and observed outcomes. Decision curve analysis (**Supplementary Figures S7A, B**) demonstrated that the combined model provided the highest net clinical benefit across a wide range of threshold probabilities compared with the clinical, radiomics, and habitat models.

### Model comparison and incremental value

Predictive score distributions are shown in **Supplementary Figure S8**, illustrating separation between responders and non-responders across different models. Comparative analyses revealed a consistent performance ranking: combined model > habitat model > clinical model > conventional radiomics model (**Figure 4A**). Furthermore, net reclassification improvement (NRI) and combined discrimination improvement (IDI) analyses demonstrated that the combined model provided significant incremental value over all single-modality models (all P < 0.01; **Supplementary Figures S9**–**S11**).

### Subgroup analysis

Subgroup analyses of the combined model were performed to evaluate its robustness across different patient subsets. The model demonstrated consistent predictive performance across major EGFR mutation subtypes (19del and L858R) and between male and female patients in both the training and validation cohorts, with comparable AUC values observed across subgroups. Notably, a slight reduction in sensitivity was observed in patients with bone metastasis compared with those without bone involvement, suggesting that tumor heterogeneity in metastatic sites may affect model performance. However, overall predictive performance remained acceptable across all subgroups. These findings indicate that the proposed model is generally robust and applicable across heterogeneous patient populations (**Supplementary Table 13**).

### Survival analysis

**Figure 5:**
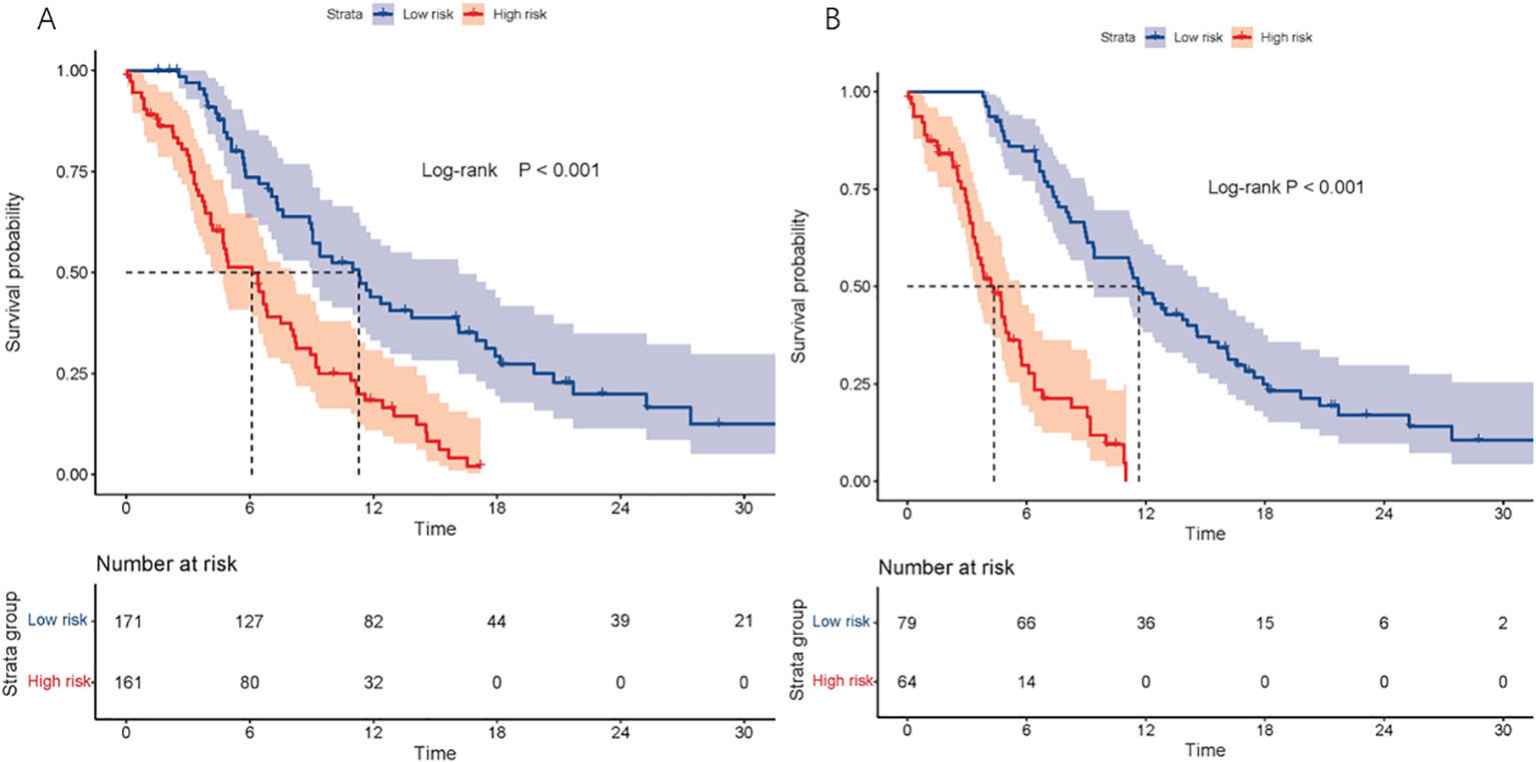
Survival analysis based on the Combined Model. Kaplan-Meier survival curves stratified by the Combined Model into low-risk and high-risk groups in the **(A)** training and **(B)** validation cohort.

Patients were stratified into high- and low-risk groups based on the model-derived cutoff determined by the Youden index. Kaplan–Meier analysis based on the combined model demonstrated clear stratification of overall survival in both cohorts (**Figure 5**). In the training cohort, the median overall survival was 11.8 months in the low-risk group and 6.1 months in the high-risk group (HR = 3.688, 95% CI: 2.244-6.06; log-rank P < 0.001). Similarly, in the validation cohort, the median overall survival was 11.9 months in the low-risk group and 4.5 months in the high-risk group (HR = 2.823, 95% CI: 2.005-3.975; log-rank P < 0.001), confirming the reproducibility of the prognostic stratification. Furthermore, multivariate Cox regression analysis demonstrated that the combined model remained an independent predictor of overall survival after adjustment for clinical variables (**Supplementary Tables 14**, **15**). These findings indicate that the combined model not only predicts treatment response but also provides robust prognostic stratification and may have potential clinical utility.

## Discussion

This study developed and validated a CT-based habitat radiomics model for predicting response to immunochemotherapy in EGFR-mutant lung adenocarcinoma following TKI resistance. The combined model demonstrated consistently superior performance across both training and external validation cohorts, significantly outperforming clinical and conventional radiomics models. It not only improved response prediction but also enabled effective survival stratification, highlighting its potential as a clinically applicable decision-support tool.

Notably, the relatively high negative predictive value (NPV) of the combined model suggests that patients predicted as non-responders are less likely to benefit from immunochemotherapy. This has important clinical implications, as it may help avoid unnecessary treatment-related toxicity and facilitate more appropriate alternative therapeutic strategies. However, this finding should be interpreted with caution, as treatment decisions should not rely solely on model predictions.

The superior performance of the combined model can be attributed to its ability to capture intratumoral spatial heterogeneity, a key biological feature associated with treatment resistance and immune response (14, 15). Unlike conventional radiomics, which treats tumors as homogeneous entities, habitat imaging decomposes tumors into distinct subregions with potentially different biological characteristics. In our study, habitat-based features consistently outperformed whole-tumor radiomics, suggesting that spatially resolved imaging phenotypes provide more informative representations of tumor behavior. The marked imbalance between sensitivity and specificity observed for the habitat model in the validation cohort also deserves consideration. Using the Youden index-optimized threshold, the habitat model achieved very high sensitivity but relatively lower specificity, suggesting that it preferentially identified responders while misclassifying a subset of non-responders. Clinically, this pattern may be advantageous for minimizing missed opportunities for potentially beneficial treatment in the post-TKI resistance setting. However, lower specificity may also increase the risk of overtreatment if the habitat model is used in isolation. This limitation further supports the value of the combined model, which achieved a more balanced overall performance.

From a biological perspective, these imaging habitats may reflect variations in the tumor microenvironment (TME) (16), including differences in cellular density, vascularization, hypoxia, and immune infiltration. Specifically, Habitat 1 may represent relatively homogeneous tumor regions characterized by more uniform gray-level distribution and lower textural complexity. Habitat 2 may correspond to relatively hypervascular or contrast-enhancing regions, potentially reflecting increased perfusion and active tumor components. Habitat 3 may represent more heterogeneous regions with greater gray-level variability and lower attenuation uniformity, which may be associated with hypoxic or necrotic areas and a more disorganized microenvironment.

At the feature level, further analysis of the most contributory habitat-derived features provides additional insight into the biological relevance of the model. The distribution differences of the two most contributory habitat features further support the biological relevance of the model. Higher SmallAreaEmphasis may reflect finer spatial heterogeneity and more fragmented tumor subregions, potentially associated with active tumor–immune interactions. In contrast, increased ZoneVariance may indicate greater structural disorganization and microenvironmental instability, which could be associated with treatment resistance. Notably, heterogeneous distribution of immune cells—such as CD8+ T cells (17, 18)—has been shown to influence response to immunotherapy, with “hot” tumors exhibiting inflamed phenotypes and “cold” tumors characterized by immune exclusion or desertification (19). The ability of habitat imaging to capture such spatial heterogeneity may explain its improved predictive performance in identifying responders and non-responders. These findings provide a plausible biological explanation for the predictive performance of the habitat model. These interpretations are hypothesis-generating and should be interpreted with caution, as no direct pathology–imaging correlation was performed in this study.

These imaging phenotypes may also be linked to different tumor immune microenvironment states. Regions with higher SmallAreaEmphasis may correspond to more heterogeneous and fragmented tumor habitats, potentially associated with immune-inflamed (“hot”) phenotypes characterized by increased immune cell infiltration. In contrast, regions with higher ZoneVariance may reflect more structurally disorganized and heterogeneous areas, which could be associated with immune-desert or immune-excluded phenotypes, as well as the spatial distribution of resistant tumor clones, hypoxia, necrosis, and aberrant angiogenesis following EGFR-TKI resistance. These interpretations are hypothesis-generating and require further validation through histopathological and molecular studies.

In contrast, the clinical model demonstrated limited predictive ability, with only EGFR mutation subtype remaining significant in multivariate analysis. This finding is consistent with previous studies indicating that conventional clinical variables alone are insufficient for predicting response to immunochemotherapy in EGFR-mutant non-small cell lung cancer (20, 21). The complexity of tumor–immune interactions likely cannot be adequately captured by traditional clinicopathological features, further supporting the need for advanced imaging-based biomarkers. This finding highlights the limited value of traditional clinical variables in predicting immunochemotherapy response in EGFR-mutant NSCLC.

The limited significance of most clinical variables in the multivariate analysis may be attributed to the relatively homogeneous baseline characteristics of the study population, potential collinearity among variables, and the inability of conventional clinical features to capture complex tumor–immune interactions in this setting.

In addition, the baseline clinical variables that differed significantly between responders and non-responders were not entirely consistent between the training and validation cohorts. This variability may reflect differences in patient composition and sample size across centers, and suggests that clinical predictors of treatment response may not be sufficiently stable across cohorts. The observed variability in baseline clinical predictors across cohorts further suggests that clinical variables alone may not provide stable predictive performance, underscoring the added value of imaging-based approaches. This interpretation is further supported by the finding that the combined model retained independent prognostic value after adjustment for clinical variables.

Importantly, the robustness of our model is supported by its consistent performance in an independent external validation cohort. In addition, rigorous feature selection procedures and cross-validation strategies were applied during model development to mitigate the risk of overfitting. These design elements enhance the generalizability and reliability of our findings.

The integration of habitat radiomics, conventional radiomics, and clinical features further improved model performance, highlighting the complementary value of multimodal data. While habitat features capture spatial heterogeneity, conventional radiomics reflects global tumor characteristics, and clinical variables provide contextual patient information. This synergistic combination enables a more comprehensive assessment of tumor behavior and treatment response (22, 23).

Compared with previous radiomics studies aiming to predict response to immunotherapy in lung cancer, our model demonstrated competitive performance. Prior studies have reported AUC values typically ranging from approximately 0.70 to 0.85 (24–26), with moderate sensitivity and specificity. In contrast, our combined model achieved an AUC of 0.890 in the external validation cohort, along with more balanced sensitivity and specificity, indicating improved overall discriminative performance and robustness. These improvements may be attributed to the incorporation of habitat-based features, which capture intratumoral spatial heterogeneity that is often overlooked in conventional whole-tumor radiomics approaches. By integrating clinical variables, conventional radiomics, and habitat-derived features, our model provides a more comprehensive characterization of tumor biology, thereby enhancing predictive performance. Nevertheless, differences in patient populations, treatment regimens, and study designs across published studies should be considered when interpreting these comparisons.

Clinically, our model has potential implications for personalized treatment decision-making. By identifying patients who are more likely to benefit from immunochemotherapy, clinicians can optimize therapeutic strategies and avoid unnecessary toxicity in non-responders. This is particularly relevant in the post-TKI resistance setting, where treatment options remain limited and outcomes are heterogeneous.

Despite these promising findings, several limitations of this study should be acknowledged. First, this was a retrospective study, which may introduce selection bias despite the use of multicenter data. Prospective studies are warranted to further validate the generalizability of our model. Second, variations in CT acquisition parameters across different centers may affect the robustness of radiomics features. Although image preprocessing and standardization were performed, residual heterogeneity may still exist. Future studies should incorporate more standardized imaging protocols or advanced harmonization techniques to further improve feature stability. Third, the biological interpretation of habitat-based features remains inferential, as no direct pathology–imaging correlation was performed. Future studies integrating imaging with histopathological and molecular analyses are needed to better validate the biological basis of these imaging phenotypes. Fourth, although an external validation cohort was included, it was derived from a single center with a relatively limited sample size, which may restrict the generalizability of the findings. Validation in larger, multi-institutional cohorts is required. Finally, the model has not yet been prospectively validated in real-world clinical settings. Prospective clinical trials are needed to assess its clinical utility and impact on treatment decision-making.

## Conclusion

In conclusion, this study developed and externally validated a CT-based habitat radiomics model for predicting response to immunochemotherapy in EGFR-mutant lung adenocarcinoma following EGFR-TKI resistance. By integrating clinical variables, conventional radiomics, and habitat-derived features, the proposed model achieved improved predictive performance compared with single-modality approaches. Importantly, the incorporation of habitat imaging enabled characterization of intratumoral spatial heterogeneity, providing additional biological insight beyond conventional whole-tumor analysis. These findings suggest that multimodal imaging-based models may offer a more comprehensive assessment of tumor behavior and treatment response. Nevertheless, given the retrospective design and limited external validation, further prospective, multi-center studies and integration with pathological and molecular data are warranted to confirm the clinical utility and biological relevance of this approach.

## Data Availability

The original contributions presented in the study are included in the article/**Supplementary Material**. Further inquiries can be directed to the corresponding author.
