# Supplementary material for: Predicting response to immunochemotherapy in EGFR-mutant lung adenocarcinoma after third-generation TKI resistance using CT radiomics-based habitat imaging
Source: Front Immunol. 2026 Jun 2;17:1785968. doi: 10.3389/fimmu.2026.1785968 (PMC13269283; doi:10.3389/fimmu.2026.1785968)
Supplement: Supplementary file 1 [file DataSheet1.docx]

Predicting Response to Immunochemotherapy in EGFR-Mutant Lung Adenocarcinoma after Third-Generation TKI Resistance Using CT Radiomics-Based Habitat Imaging

**Supplementary materials**

1. **Supplementary Data**

Our methodology for delineating tumor Intratumor Heterogeneity regions was multi-faceted and involved several intricate steps:

1. Advanced Superpixel Segmentation: Utilizing the SLIC algorithm within the scikit-learn framework, we initially segmented each tumor's Region of Interest (ROI) into 100 subregions. The segmentation was fine-tuned with a compactness parameter set at 10.0, balancing color similarity and spatial proximity.

The SLIC algorithm is a method for superpixel segmentation. It works by clustering pixels in the image based on their color similarity and proximity in the image space. The key formula for SLIC is the distance measure, which combines color and spatial proximity:


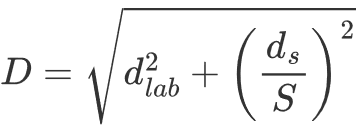


- D is the combined distance measure.

- d is the Euclidean distance in color space (Lab color space).

- ds is the Euclidean distance in the image plane.

- S is the grid interval or the size of the superpixel.

1. **Supplementary Tables and Figures**

**2.1 Supplementary Tables**

**Supplementary Table S1.** CT scanning parameters of the two centers.

| Parameters | Training cohort (Center A) | External validation cohort (Center B) |
| --- | --- | --- |
| Manufacturer | Siemens | GE |
| Acquisition type | Helical | Helical |
| Tube voltage | 120 kVp | 120 kVp |
| Tube current | Automatic tube current modulation | Automatic tube current modulation |
| Pitch | 0.75–1.0 | 1.2–1.5 |
| Collimation | 0.6 mm | 0.6 mm |
| Matrix | 512 × 512 | 512 × 512 |
| Pixel spacing | 0.75 × 0.75 mm | 0.6–0.96 × 0.6–0.96 mm |
| Reconstructed slice thickness | 0.625 mm | 0.625 mm |

**Supplementary Table S2.** Categorization of Extracted Features (Whole Tumor).

| **Features** | Counts |
| --- | --- |
| First-order | N=360 |
| shape | N=14 |
| grey level co-occurrence matrix (GLCM) | N=440 |
| grey level size zone matrix (GLSZM) | N=320 |
| neighborhood gray-tone difference matrix (NGTDM) | N=100 |
| grey level run length matrix (GLRLM) | N=320 |
| gray level dependence matrix (GLDM) | N=280 |

**Supplementary Table S3.** The types and quantities of imaging features of habitat sub-regions.

| Feature name | Counts |
| --- | --- |
| firstorder | 1080 |
| glcm | 1320 |
| gldm | 840 |
| glrlm | 960 |
| glszm | 960 |
| ngtdm | 300 |
| shape | 42 |

**Supplementary Table S4.** Coefficients of features selected by LASSO regression for the ROI-based and conventional radiomics models.

| Model | Type | Feature / Term | Coefficient |
| --- | --- | --- | --- |
| ROI-based model | Radiomics feature | intra_lbp_3D_k_glszm_HighGrayLevelZoneEmphasis_CT | 0.00077 |
| ROI-based model | Radiomics feature | intra_lbp_3D_m2_ngtdm_Complexity_CT | 0.046938 |
| ROI-based model | Radiomics feature | intra_wavelet_HHH_firstorder_RootMeanSquared_CT | -0.000227 |
| ROI-based model | Radiomics feature | intra_wavelet_LHH_firstorder_Median_CT | -0.017012 |
| ROI-based model | Radiomics feature | intra_wavelet_LLH_glcm_ClusterShade_CT | -0.018693 |
| Radiomics model | Constant | constant | 0.34337349 |
| Radiomics model | Exponential feature | exponential_firstorder_Skewness_h2 | 0.043627 |
| Radiomics model | NGLDM feature | original_ngtdm_Busyness_h3 | 0.000168 |
| Radiomics model | GLDM feature | squareroot_gldm_LargeDependenceLowGrayLevelEmphasis_h2 | 0.042005 |
| Radiomics model | LBP feature | lbp_3D_m1_firstorder_Range_h1 | 0.019028 |
| Radiomics model | LBP feature | lbp_3D_m1_firstorder_Maximum_h3 | 0.007453 |
| Radiomics model | Exponential feature | exponential_ngtdm_Busyness_h3 | 0.042127 |
| Radiomics model | GLRLM feature | logarithm_glrlm_LongRunLowGrayLevelEmphasis_h1 | -0.006482 |
| Radiomics model | GLCM feature | squareroot_glcm_Contrast_h2 | -0.010393 |
| Radiomics model | GLSZM feature | wavelet_LLL_glszm_ZoneVariance_h3 | -0.050116 |
| Radiomics model | GLSZM feature | wavelet_LHH_glszm_SmallAreaEmphasis_h3 | 0.050656 |
| Radiomics model | NGLDM feature | wavelet_HLL_ngtdm_Busyness_h3 | 0.011538 |
| Radiomics model | GLDM feature | squareroot_gldm_GrayLevelVariance_h3 | 0.015941 |
| Radiomics model | GLDM feature | exponential_gldm_SmallDependenceHighGrayLevelEmphasis_h2 | 0.008562 |
| Radiomics model | GLRLM feature | log_sigma_2_0_mm_3D_glrlm_RunEntropy_h1 | -0.027436 |
| Radiomics model | GLCM feature | wavelet_LLL_glcm_Contrast_h3 | -0.016502 |
| Radiomics model | LBP feature | lbp_3D_k_firstorder_InterquartileRange_h2 | -0.013802 |

**Supplementary Table S4(continued).**

| Radiomics model | LBP feature | lbp_3D_m2_firstorder_Minimum_h3 | -0.00308 |
| --- | --- | --- | --- |
| Radiomics model | LBP feature | lbp_3D_k_firstorder_Minimum_h1 | -0.010554 |
| Radiomics model | NGLDM feature | log_sigma_2_0_mm_3D_ngtdm_Strength_h3 | 0.003756 |

**Supplementary Table S5.** Baseline characteristics stratified by treatment response in the training cohort.

|  | ALL N=332 | No-response N=218 | Response N=114 | P value |
| --- | --- | --- | --- | --- |
| Sex |  |  |  | 1 |
| Female | 212 (63.86%) | 139 (63.76%) | 73 (64.04%) |  |
| Male | 120 (36.14%) | 79 (36.24%) | 41 (35.96%) |  |
| Age | 62.00 (55.00,69.25) | 63.00 (56.00,70.00) | 58.00 (53.00,68.00) | 0.004 |
| T stage |  |  |  | <0.001 |
| T1 | 118 (35.54%) | 111 (50.92%) | 7 (6.14%) |  |
| T2 | 77 (23.19%) | 55 (25.23%) | 22 (19.30%) |  |
| T3 | 14 (4.22%) | 9 (4.13%) | 5 (4.39%) |  |
| T4 | 123 (37.05%) | 43 (19.72%) | 80 (70.18%) |  |
| N stage |  |  |  | <0.001 |
| N0 | 57 (17.17%) | 47 (21.56%) | 10 (8.77%) |  |
| N1 | 102 (30.72%) | 101 (46.33%) | 1 (0.88%) |  |
| N2 | 80 (24.10%) | 37 (16.97%) | 43 (37.72%) |  |
| N3 | 93 (28.01%) | 33 (15.14%) | 60 (52.63%) |  |
| Smoking status |  |  |  | 0.477 |
| No | 244 (73.49%) | 157 (72.02%) | 87 (76.32%) |  |
| Yes | 88 (26.51%) | 61 (27.98%) | 27 (23.68%) |  |
| Bone metastases |  |  |  | <0.001 |
| No | 204 (61.45%) | 154 (70.64%) | 50 (43.86%) |  |
| Yes | 128 (38.55%) | 64 (29.36%) | 64 (56.14%) |  |
| Liver metastases |  |  |  | 0.221 |
| No | 299 (90.06%) | 200 (91.74%) | 99 (86.84%) |  |
| Yes | 33 (9.94%) | 18 (8.26%) | 15 (13.16%) |  |
|  |  |  |  |  |
| **Supplementary Table S5. (Continued).** | | | | |
| Pleural effusion |  |  |  | 0.503 |
| No | 222 (66.87%) | 149 (68.35%) | 73 (64.04%) |  |
| Yes | 110 (33.13%) | 69 (31.65%) | 41 (35.96%) |  |
| Pulmonary metastasis |  |  |  | 0.130 |
| No | 226 (68.07%) | 155 (71.10%) | 71 (62.28%) |  |
| Yes | 106 (31.93%) | 63 (28.90%) | 43 (37.72%) |  |
| Distant lymph node metastasis |  |  |  | 0.163 |
| No | 274 (82.53%) | 185 (84.86%) | 89 (78.07%) |  |
| Yes | 58 (17.47%) | 33 (15.14%) | 25 (21.93%) |  |
| EGFR mutations |  |  |  | 0.001 |
| 19del | 138 (41.57%) | 85 (38.99%) | 53 (46.49%) |  |
| 21 L858R | 126 (37.95%) | 75 (34.40%) | 51 (44.74%) |  |
| Other | 68 (20.48%) | 58 (26.61%) | 10 (8.77%) |  |
| Primary site |  |  |  | 0.272 |
| Left | 145 (43.67%) | 90 (41.28%) | 55 (48.25%) |  |
| Right | 187 (56.33%) | 128 (58.72%) | 59 (51.75%) |  |

**Supplementary Table S6.** Baseline characteristics stratified by treatment response in the validation cohort.

|  | ALL N=143 | No-response N=100 | Response N=43 | P value |
| --- | --- | --- | --- | --- |
| Sex |  |  |  | 0.358 |
| Female | 96 (67.13%) | 70 (70.00%) | 26 (60.47%) |  |
| Male | 47 (32.87%) | 30 (30.00%) | 17 (39.53%) |  |
| Age | 63.00 (56.00,68.50) | 64.00 (56.75,70.00) | 58.00 (54.00,64.50) | 0.003 |
| T stage |  |  |  | <0.001 |
| T1 | 57 (39.86%) | 54 (54.00%) | 3 (6.98%) |  |
| T2 | 39 (27.27%) | 26 (26.00%) | 13 (30.23%) |  |
| T3 | 8 (5.59%) | 5 (5.00%) | 3 (6.98%) |  |
| T4 | 39 (27.27%) | 15 (15.00%) | 24 (55.81%) |  |
| N stage |  |  |  | <0.001 |
| N0 | 35 (24.48%) | 31 (31.00%) | 4 (9.30%) |  |
| N1 | 39 (27.27%) | 38 (38.00%) | 1 (2.33%) |  |
| N2 | 29 (20.28%) | 16 (16.00%) | 13 (30.23%) |  |
| N3 | 40 (27.97%) | 15 (15.00%) | 25 (58.14%) |  |
| Smoking status |  |  |  | 0.803 |
| No | 110 (76.92%) | 78 (78.00%) | 32 (74.42%) |  |
| Yes | 33 (23.08%) | 22 (22.00%) | 11 (25.58%) |  |
| Bone metastases |  |  |  | 0.006 |
| No | 89 (62.24%) | 70 (70.00%) | 19 (44.19%) |  |
| Yes | 54 (37.76%) | 30 (30.00%) | 24 (55.81%) |  |
| Liver metastases |  |  |  | 0.148 |
| No | 128 (89.51%) | 92 (92.00%) | 36 (83.72%) |  |
| Yes | 15 (10.49%) | 8 (8.00%) | 7 (16.28%) |  |

| **Supplementary Table S6 (Continued).** | | | | |
| --- | --- | --- | --- | --- |
| Pleural effusion |  |  |  | 0.418 |
| No | 91 (63.64%) | 61 (61.00%) | 30 (69.77%) |  |
| Yes | 52 (36.36%) | 39 (39.00%) | 13 (30.23%) |  |
| Pulmonary metastasis |  |  |  | 0.037 |
| No | 99 (69.23%) | 75 (75.00%) | 24 (55.81%) |  |
| Yes | 44 (30.77%) | 25 (25.00%) | 19 (44.19%) |  |
| Distant lymph node metastasis |  |  |  | 1 |
| No | 114 (79.72%) | 80 (80.00%) | 34 (79.07%) |  |
| Yes | 29 (20.28%) | 20 (20.00%) | 9 (20.93%) |  |
| EGFR mutations |  |  |  | 0.141 |
| 19del | 54 (37.76%) | 33 (33.00%) | 21 (48.84%) |  |
| 21 L858R | 62 (43.36%) | 45 (45.00%) | 17 (39.53%) |  |
| Other | 27 (18.88%) | 22 (22.00%) | 5 (11.63%) |  |
| Primary site |  |  |  | 0.013 |
| Left | 56 (39.16%) | 32 (32.00%) | 24 (55.81%) |  |
| Right | 87 (60.84%) | 68 (68.00%) | 19 (44.19%) |  |

**Supplementary Table S7.** Univariate and Multivariate Logistic Regression Analysis of Risk Factors for immunochemotherapy Response.

| Parameter | Univariate analysis |  | | Multivariate analysis | |  | |  |
| --- | --- | --- | --- | --- | --- | --- | --- | --- |
|  | OR (95% CI) | *P* value | | OR (95% CI) | | *P* value | |  |
| Age | 0.989 (95% CI: 0.986–0.992) | <0.05 | |  | |  | |  |
| Sex |  | <0.05 | |  | | 0.349 | |  |
| Female | Reference |  | | Reference | |  | |  |
| Male | 0.519 (95% CI: 0.378–0.712) |  | | 1.361 (95% CI: 0.792–2.337) | |  | |  |
| Pleural effusion |  | <0.05 | |  | | 0.342 | |  |
| No | Reference |  | | Reference | |  | |  |
| Yes | 0.594 (95% CI: 0.430–0.822) |  | | 1.266 (95% CI: 0.841–1.906) | |  | |  |
| Smoking status |  | <0.05 | |  | | 0.196 | |  |
| No | Reference |  | |  | |  | |  |
| Yes | 0.443 (95% CI: 0.303–0.647) |  | | 0.626 (95% CI: 0.345–1.135) | |  | |  |
| Primary site |  | <0.05 | |  | | 0.892 | |  |
| Left | Reference |  | | Reference | |  | |  |
| Right | 0.666 (95% CI: 0.592–0.750) |  | | 0.971 (95% CI: 0.685–1.380) | |  | |  |
| EGFR mutations |  | <0.05 | |  | | <0.05 | |  |
| 19del | Reference |  | | Reference | |  | |  |
| 21 L858R | 0.725 (95% CI: 0.559–0.942) |  | | 0.725 (95% CI: 0.559–0.942) | |  | |  |
| Pulmonary metastasis |  | 0.054 | |  | |  | |  |
| No | Reference |  | |  | |  | |  |
| Yes | 0.683 (95% CI: 0.493–0.945) |  | |  | |  | |  |
|  |  |  | |  | |  | |  |
| **Supplementary Table S7 (continued).** | |  | |  | |  | |  |
| Brain metastasis |  |  | |  | |  | |  |
| No | Reference |  | |  | |  | |  |
| Yes | 0.758 (95% CI: 0.490–1.171) | |  | |  | |  | |
| Liver metastases |  | | 0.602 | |  | |  | |
| No | Reference | |  | |  | |  | |
| Yes | 0.833 (95% CI: 0.469–1.481) | |  | |  | |  | |
| T stage |  | | 0.498 | |  | |  | |
| T1 | Reference | |  | |  | |  | |
| T2 | 1.310 (95% CI: 0.955–1.797) | | 0.094 | |  | |  | |
| T3 | 1.422 (95% CI: 1.040–1.946) | | 0.028 | |  | |  | |
| T4 | 1.424 (95% CI: 1.056–1.922) | | 0.021 | |  | |  | |
| N stage |  | | 0.844 | |  | |  | |
| N0 | Reference | |  | |  | |  | |
| N1 | 1.249 (95% CI: 0.841–1.855) | | 0.270 | |  | |  | |
| N2 | 1.348 (95% CI: 1.066–1.705) | | 0.013 | |  | |  | |
| N3 | 1.187 (95% CI: 0.918–1.535) | | 0.190 | |  | |  | |
| Bone metastases |  | |  | |  | |  | |
| No | Reference | | 1 | |  | |  | |
| Yes | 1.000 (95% CI: 0.748–1.338) | |  | |  | |  | |

**Supplementary Table S8.** Performance Comparison of Machine Learning Algorithms for Predicting Response Using Clinical Features.

| Model name | Accuracy | AUC | 95% CI | Sensitivity | Specificity | PPV | NPV | Precision | Recall | F1 | Threshold | Cohort |
| --- | --- | --- | --- | --- | --- | --- | --- | --- | --- | --- | --- | --- |
| SVM | 0.660 | 0.609 | 0.542–0.676 | 0.439 | 0.775 | 0.505 | 0.725 | 0.505 | 0.439 | 0.469 | 0.591 | training |
| SVM | 0.650 | 0.601 | 0.493–0.708 | 0.488 | 0.720 | 0.429 | 0.766 | 0.429 | 0.488 | 0.457 | 0.585 | validation |
| RandomForest | 0.705 | 0.740 | 0.686–0.794 | 0.570 | 0.775 | 0.570 | 0.775 | 0.570 | 0.570 | 0.570 | 0.530 | training |
| RandomForest | 0.650 | 0.678 | 0.581–0.775 | 0.651 | 0.650 | 0.444 | 0.812 | 0.444 | 0.651 | 0.528 | 0.507 | validation |
| ExtraTrees | 0.675 | 0.718 | 0.662–0.774 | 0.667 | 0.679 | 0.521 | 0.796 | 0.521 | 0.667 | 0.585 | 0.506 | training |
| ExtraTrees | 0.622 | 0.697 | 0.603–0.790 | 0.721 | 0.580 | 0.425 | 0.829 | 0.425 | 0.721 | 0.534 | 0.513 | validation |
| XGBoost | 0.693 | 0.680 | 0.621–0.739 | 0.386 | 0.853 | 0.579 | 0.727 | 0.579 | 0.386 | 0.463 | 0.615 | training |
| XGBoost | 0.671 | 0.680 | 0.585–0.774 | 0.512 | 0.740 | 0.458 | 0.779 | 0.458 | 0.512 | 0.484 | 0.503 | validation |
| LightGBM | 0.669 | 0.672 | 0.613–0.730 | 0.430 | 0.794 | 0.521 | 0.727 | 0.521 | 0.430 | 0.471 | 0.554 | training |
| LightGBM | 0.685 | 0.672 | 0.575–0.768 | 0.465 | 0.780 | 0.476 | 0.772 | 0.476 | 0.465 | 0.471 | 0.544 | validation |

AUC, Area Under the (ROC) Curve; PPV, Positive Predictive Value; NPV, Negative Predictive Value.

**Supplementary Table S9.** Performance Comparison of Machine Learning Algorithms for Predicting immunochemotherapy response Status Using Radiomics Signature.

| Model name | Accuracy | AUC | 95% CI | Sensitivity | Specificity | PPV | NPV | Precision | Recall | F1 | Threshold | Cohort |
| --- | --- | --- | --- | --- | --- | --- | --- | --- | --- | --- | --- | --- |
| VM | 0.605 | 0.616 | 0.553–0.679 | 0.614 | 0.601 | 0.446 | 0.749 | 0.446 | 0.614 | 0.517 | 0.526 | training |
| SVM | 0.531 | 0.615 | 0.520–0.709 | 0.837 | 0.400 | 0.375 | 0.851 | 0.375 | 0.837 | 0.518 | 0.526 | validation |
| RandomForest | 0.578 | 0.656 | 0.594–0.718 | 0.737 | 0.495 | 0.433 | 0.783 | 0.433 | 0.737 | 0.545 | 0.479 | training |
| RandomForest | 0.678 | 0.657 | 0.563–0.751 | 0.605 | 0.710 | 0.473 | 0.807 | 0.473 | 0.605 | 0.531 | 0.530 | validation |
| ExtraTrees | 0.593 | 0.661 | 0.601–0.722 | 0.667 | 0.555 | 0.439 | 0.761 | 0.439 | 0.667 | 0.530 | 0.454 | training |
| ExtraTrees | 0.615 | 0.657 | 0.561–0.752 | 0.767 | 0.550 | 0.423 | 0.846 | 0.423 | 0.767 | 0.545 | 0.460 | validation |
| XGBoost | 0.648 | 0.659 | 0.597–0.721 | 0.430 | 0.761 | 0.485 | 0.719 | 0.485 | 0.430 | 0.456 | 0.531 | training |
| XGBoost | 0.622 | 0.647 | 0.554–0.740 | 0.628 | 0.620 | 0.415 | 0.795 | 0.415 | 0.628 | 0.500 | 0.481 | validation |
| LightGBM | 0.654 | 0.660 | 0.598–0.722 | 0.447 | 0.761 | 0.495 | 0.725 | 0.495 | 0.447 | 0.470 | 0.528 | training |
| LightGBM | 0.629 | 0.658 | 0.566–0.751 | 0.721 | 0.590 | 0.431 | 0.831 | 0.431 | 0.721 | 0.539 | 0.487 | validation |

AUC, Area Under the (ROC) Curve; PPV, Positive Predictive Value; NPV, Negative Predictive Value.

**Supplementary Table S10.** Comparison of model performance among different machine learning algorithms based on habitat imaging.

| Model name | Accuracy | AUC | 95% CI | Sensitivity | Specificity | PPV | NPV | Precision | Recall | F1 | Threshold | Cohort |
| --- | --- | --- | --- | --- | --- | --- | --- | --- | --- | --- | --- | --- |
| SVM | 0.750 | 0.813 | 0.765–0.861 | 0.798 | 0.725 | 0.603 | 0.873 | 0.603 | 0.798 | 0.687 | 0.447 | training |
| SVM | 0.755 | 0.750 | 0.660–0.839 | 0.651 | 0.800 | 0.583 | 0.842 | 0.583 | 0.651 | 0.615 | 0.477 | validation |
| RandomForest | 0.822 | 0.881 | 0.844–0.918 | 0.798 | 0.835 | 0.717 | 0.888 | 0.717 | 0.798 | 0.755 | 0.515 | training |
| RandomForest | 0.650 | 0.783 | 0.707 – 0.860 | 0.814 | 0.580 | 0.455 | 0.879 | 0.455 | 0.814 | 0.583 | 0.439 | validation |
| ExtraTrees | 0.756 | 0.880 | 0.844–0.917 | 0.895 | 0.683 | 0.596 | 0.925 | 0.596 | 0.895 | 0.716 | 0.484 | training |
| ExtraTrees | 0.608 | 0.720 | 0.634–0.806 | 0.814 | 0.520 | 0.422 | 0.867 | 0.422 | 0.814 | 0.556 | 0.459 | validation |
| XGBoost | 0.786 | 0.845 | 0.801–0.889 | 0.719 | 0.821 | 0.678 | 0.848 | 0.678 | 0.719 | 0.698 | 0.538 | training |
| XGBoost | 0.741 | 0.822 | 0.751–0.894 | 0.814 | 0.710 | 0.547 | 0.899 | 0.547 | 0.814 | 0.654 | 0.479 | validation |
| LightGBM | 0.816 | 0.900 | 0.866–0.934 | 0.798 | 0.826 | 0.705 | 0.887 | 0.705 | 0.798 | 0.749 | 0.512 | training |
| LightGBM | 0.748 | 0.874 | 0.819–0.929 | 0.977 | 0.650 | 0.545 | 0.985 | 0.545 | 0.977 | 0.700 | 0.409 | validation |

**Supplementary Table S11. Comparison of the performance of four models: clinical, radiomics, habitat, and combined.**

| Signature | Accuracy | AUC | 95% CI | Sensitivity | Specificity | PPV | NPV | Precision | Recall | F1 | Threshold | Cohort |
| --- | --- | --- | --- | --- | --- | --- | --- | --- | --- | --- | --- | --- |
| Clinical | 0.675 | 0.718 | 0.663–0.774 | 0.667 | 0.679 | 0.521 | 0.796 | 0.521 | 0.667 | 0.585 | 0.506 | training |
| Radiomics | 0.654 | 0.660 | 0.598–0.722 | 0.447 | 0.761 | 0.495 | 0.725 | 0.495 | 0.447 | 0.470 | 0.528 | training |
| Habitat | 0.816 | 0.900 | 0.866–0.934 | 0.798 | 0.826 | 0.705 | 0.887 | 0.705 | 0.798 | 0.749 | 0.512 | training |
| Combined | 0.798 | 0.904 | 0.871–0.937 | 0.895 | 0.748 | 0.650 | 0.931 | 0.650 | 0.895 | 0.753 | 0.252 | training |
| Clinical | 0.622 | 0.697 | 0.603–0.790 | 0.721 | 0.580 | 0.425 | 0.829 | 0.425 | 0.721 | 0.534 | 0.513 | validation |
| Radiomics | 0.629 | 0.658 | 0.566–0.751 | 0.721 | 0.590 | 0.431 | 0.831 | 0.431 | 0.721 | 0.539 | 0.487 | validation |
| Habitat | 0.748 | 0.874 | 0.819–0.929 | 0.977 | 0.650 | 0.545 | 0.985 | 0.545 | 0.977 | 0.700 | 0.409 | validation |
| Combined | 0.769 | 0.890 | 0.838–0.942 | 0.860 | 0.730 | 0.578 | 0.924 | 0.578 | 0.860 | 0.692 | 0.218 | validation |

AUC, Area Under the (ROC) Curve; PPV, Positive Predictive Value; NPV, Negative Predictive Value.

**Supplementary Table S12.** Pairwise comparison of model performance using the DeLong test.

| Comparison | AUC difference | 95% CI | Z value | P value |
| --- | --- | --- | --- | --- |
| Combined vs Clinical | 0.287 | 95% CI: 0.167–0.408 | 4.64 | < 0.001 |
| Combined vs Radiomics | 0.276 | 95% CI: 0.181–0.375 | 5.61 | < 0.001 |
| Combined vs Habitat | 0.017 | 95% CI: −0.007–0.041 | 1.38 | 0.158 |
| Clinical vs Radiomics | −0.011 | 95% CI: −0.164–0.137 | −0.15 | 0.589 |
| Clinical vs Habitat | −0.270 | 95% CI: −0.394–−0.148 | −4.28 | < 0.001 |
| Radiomics vs Habitat | −0.259 | 95% CI: −0.362–−0.162 | −5.11 | < 0.001 |

Abbreviations: AUC, area under the curve; CI, confidence interval. Pairwise comparisons between models were performed using the DeLong test.

**Supplementary Table S13.** Performance of the combined model across clinical subgroups in the training and validation cohorts.

| Subgroup | **Training (N)** | AUC | Sensitivity | Specificity | **Validation (N)** | AUC | Sensitivity | Specificity |
| --- | --- | --- | --- | --- | --- | --- | --- | --- |
| EGFR 19del | 138 | 0.908 | 0.755 | 0.906 | 54 | 0.873 | 1 | 0.625 |
| EGFR L858R | 126 | 0.896 | 0.750 | 0.897 | 62 | 0.864 | 1 | 0.659 |
| Male | 212 | 0.910 | 0.926 | 0.750 | 96 | 0.896 | 0.931 | 0.716 |
| Female | 120 | 0.918 | 0.914 | 0.812 | 47 | 0.901 | 0.937 | 0.806 |
| Bone metastasis (Yes) | 128 | 0.925 | 0.977 | 0.741 | 54 | 0.887 | 0.692 | 0.951 |
| Bone metastasis (No) | 204 | 0.896 | 0.808 | 0.841 | 89 | 0.899 | 1 | 0.633 |

**Supplementary Table S14.** Univariate and multivariate Cox regression analyses of clinical variables and model-derived risk groups for overall survival in the training cohorts.

| Training set | Univariate analysis | | | Multivariate analysis | | |
| --- | --- | --- | --- | --- | --- | --- |
| **characteristics** | **HR** | **95%CI** | **P value** | **HR** | **95%CI** | **P value** |
| Age | 1.047 | 1.021–1.074 | <0.001 | 1.038 | 1.011–1.066 | 0.005 |
| Model-derived risk score |  |  |  |  |  |  |
| Low risk | Reference |  |  | Reference |  |  |
| High risk | 3.648 | 2.292–5.806 | <0.001 | 3.688 | 2.244–6.06 | <0.001 |
| Liver metastasis |  |  |  |  |  |  |
| No | Reference |  |  | Reference |  |  |
| Yes | 2.264 | 1.416–3.619 | <0.001 | 1.810 | 1.062–3.085 | 0.029 |
| Bone metastasis |  |  |  |  |  |  |
| No | Reference |  |  | Reference |  |  |
| Yes | 2.507 | 1.605–3.915 | <0.001 | 1.852 | 1.150–2.983 | 0.011 |
| Pleural effusion |  |  |  |  |  |  |
| No | Reference |  |  |  |  |  |
| Yes | 1.040 | 0.662–1.633 | 0.864 |  |  |  |
| N stage |  |  |  |  |  |  |
| N0 | Reference |  |  |  |  |  |
| N1 | 0.778 | 0.377–1.606 | 0.498 |  |  |  |
| N2 | 0.885 | 0.478–1.639 | 0.697 |  |  |  |
| N3 | 0.961 | 0.516–1.788 | 0.900 |  |  |  |
| Pulmonary metastasis |  |  |  |  |  |  |
| No | Reference |  |  |  |  |  |
| Yes | 0.928 | 0.604–1.426 | 0.734 |  |  |  |

**Supplementary Table S14. (continued)**

| EGFR mutations | Univariate analysis | | | Multivariate analysis | | |
| --- | --- | --- | --- | --- | --- | --- |
| 19 del | Reference |  |  |  |  |  |
| 21 L858R | 1.180 | 0.748–1.862 | 0.475 |  |  |  |
| other | 1.125 | 0.536–2.363 | 0.755 |  |  |  |
| T stage |  |  |  |  |  |  |
| T1 | Reference |  |  |  |  |  |
| T2 | 0.918 | 0.506–1.664 | 0.778 |  |  |  |
| T3 | 1.094 | 0.612–1.954 | 0.761 |  |  |  |
| T4 | 1.099 | 0.553–2.184 | 0.788 |  |  |  |
| Primary site |  |  |  |  |  |  |
| Left | Reference |  |  |  |  |  |
| Right | 0.758 | 0.495–1.162 | 0.203 |  |  |  |
| Smoking Status |  |  |  |  |  |  |
| No | Reference |  |  |  |  |  |
| Yes | 1.243 | 0.867–1.783 | 0.237 |  |  |  |
| Sex |  |  |  |  |  |  |
| Female | Reference |  |  |  |  |  |
| Male | 0.940 | 0.573–1.541 | 0.805 |  |  |  |

**Supplementary Table S15.** Univariate and multivariate Cox regression analyses of clinical variables and model-derived risk groups for overall survival in the validation cohorts.

|  | Univariate analysis |  |  | Multivariate analysis |  |  |
| --- | --- | --- | --- | --- | --- | --- |
| **characteristics** | **HR** | **95%CI** | **P value** | **HR** | **95%CI** | **P value** |
| Age | 1.037 | 1.019–1.056 | <0.001 | 1.023 | 1.004–1.042 | 0.018 |
| Liver metastasis |  |  |  |  |  |  |
| No | Reference |  |  | Reference |  |  |
| Yes | 2.577 | 1.809–3.672 | <0.001 | 1.806 | 1.214–2.687 | 0.004 |
| Model-derived risk score |  |  |  |  |  |  |
| Low risk | Reference |  |  |  |  |  |
| High risk | 3.336 | 2.383–4.672 | <0.001 | 2.823 | 2.005–3.975 | <0.001 |
| Bone metastasis | 2.532 | 1.817–3.527 | <0.001 | 1.528 | 1.050–2.223 | 0.027 |
| No | Reference |  |  |  |  |  |
| Yes |  |  |  |  |  |  |
| Pleural effusion |  |  |  |  |  |  |
| No | Reference |  |  |  |  |  |
| Yes | 1.012 | 0.726–1.410 | 0.944 |  |  |  |
| N stage |  |  |  |  |  |  |
| N0 | Reference |  |  |  |  |  |
| N1 | 0.894 | 0.524–1.525 | 0.680 |  |  |  |
| N2 | 0.992 | 0.626–1.573 | 0.974 |  |  |  |
| N3 | 0.910 | 0.562–1.475 | 0.703 |  |  |  |

**Supplementary Table S15(continued).**

| Pulmonary metastasis |  |  |  |  |  |  |
| --- | --- | --- | --- | --- | --- | --- |
| No | Reference |  |  |  |  |  |
| Yes | 0.802 | 0.583–1.103 | 0.175 |  |  |  |
| EGFR mutations |  |  |  |  |  |  |
| 19 del | Reference |  |  |  |  |  |
| 21 L858R | 0.996 | 0.713–1.393 | 0.984 |  |  |  |
| other | 1.069 | 0.611–1.873 | 0.815 |  |  |  |
| T stage |  |  |  |  |  |  |
| T1 | Reference |  |  |  |  |  |
| T2 | 0.717 | 0.457–1.125 | 0.147 |  |  |  |
| T3 | 1.218 | 0.795–1.864 | 0.365 |  |  |  |
| T4 | 1.313 | 0.809–2.133 | 0.271 |  |  |  |
| Primary site |  |  |  |  |  |  |
| Left | Reference |  |  |  |  |  |
| Right | 0.987 | 0.719–1.357 | 0.938 |  |  |  |
| Smoking Status |  |  |  |  |  |  |
| No | Reference |  |  |  |  |  |
| Yes | 1.422 | 0.884–2.286 | 0.147 |  |  |  |
| Sex |  |  |  |  |  |  |
| Female | Reference |  |  |  |  |  |
| Male | 0.889 | 0.604–1.307 | 0.549 |  |  |  |

**2.2 Supplementary Figures**

**
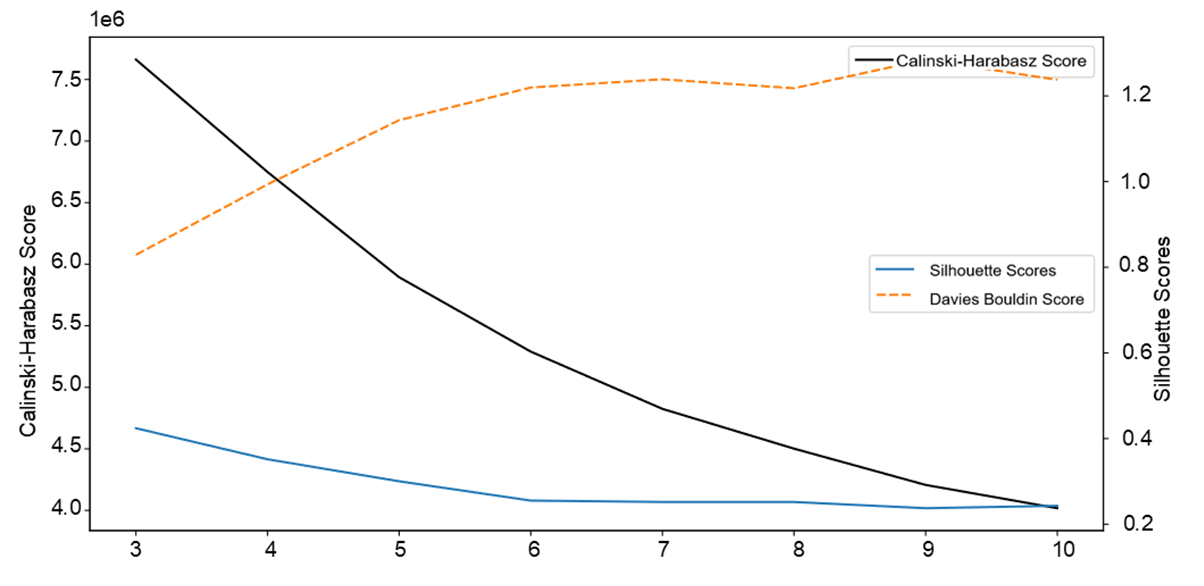
Supplementary Figure S1.** Determination of the optimal cluster number (k) for habitat segmentation using the Calinski-Harabasz (CH) index.​ The peak CH score at k=3 indicates the optimal separation of imaging habitats, justifying the choice of three distinct subregions for subsequent analysis.

**
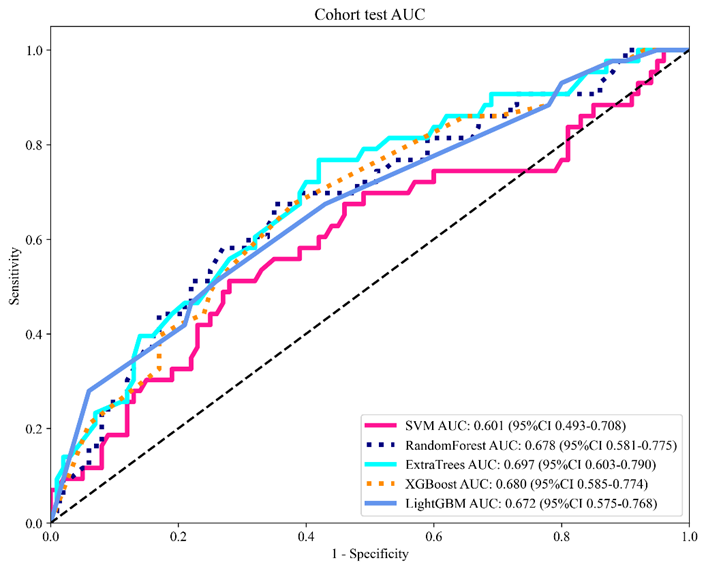

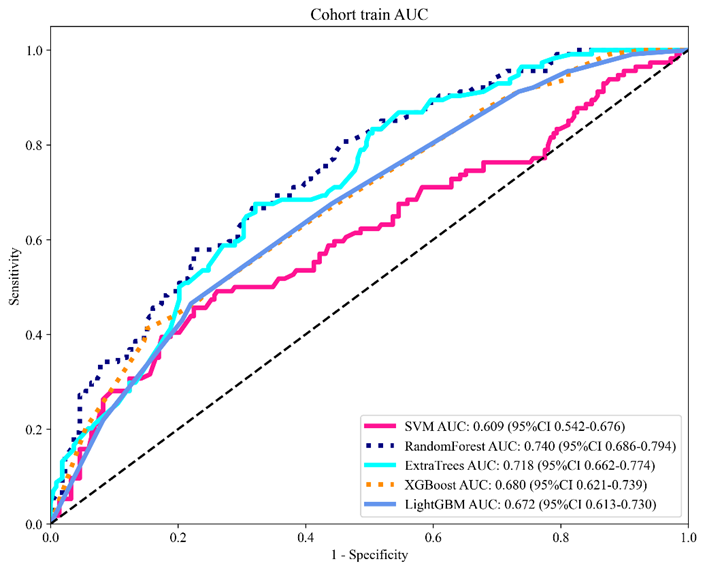
Supplementary Figure S2.**

Receiver operating characteristic (ROC) curves of different models based on clinical signature.

(A) Training cohort.

(B) Validation cohort.


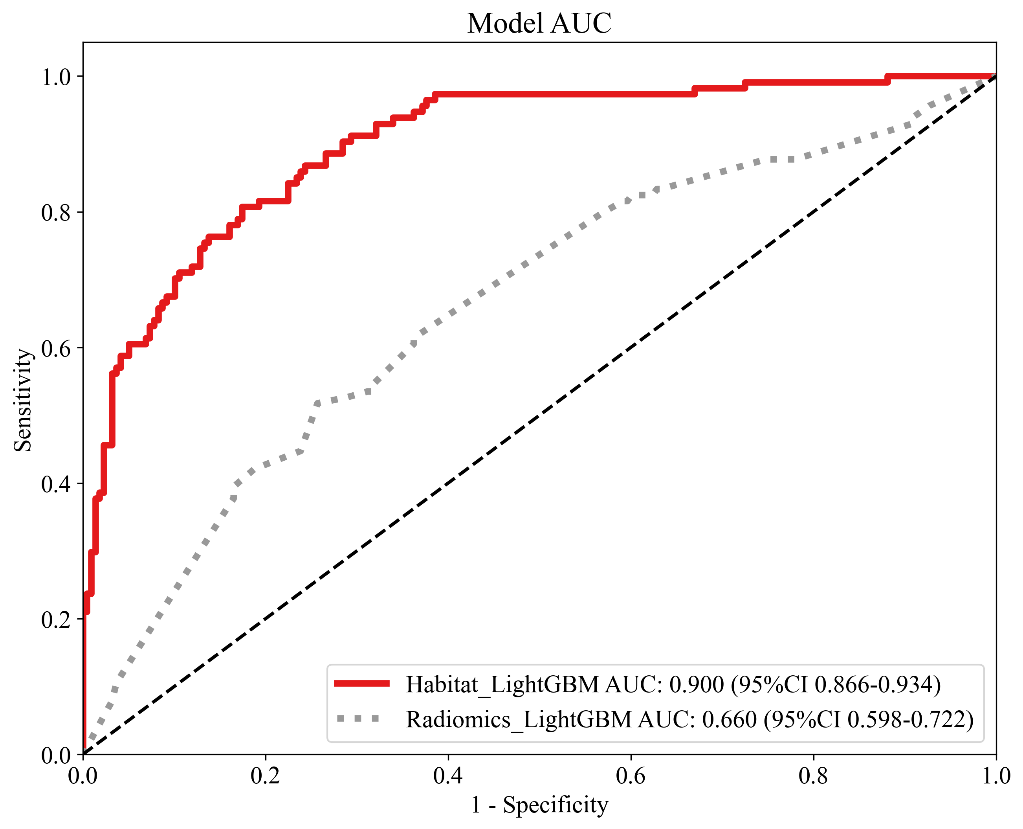

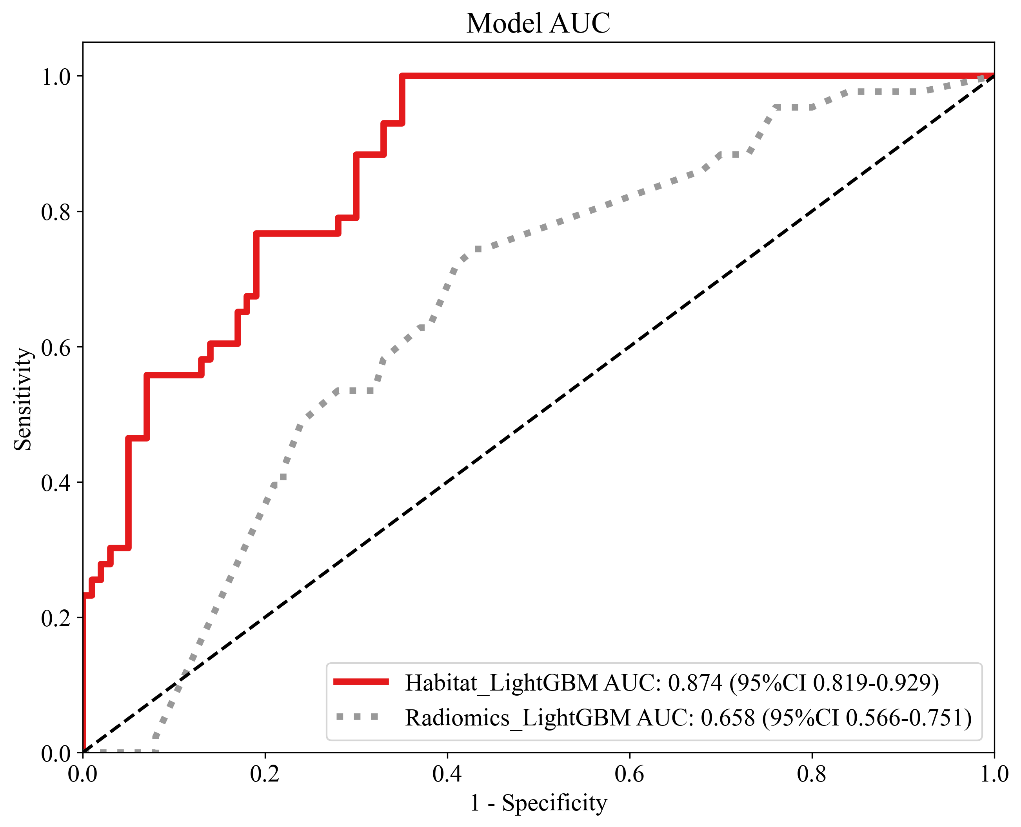


**Supplementary Figure S3.**

Receiver operating characteristic (ROC) curves of different models based on radiomics signature.

(A) Training cohort.

(B) Validation cohort.

**
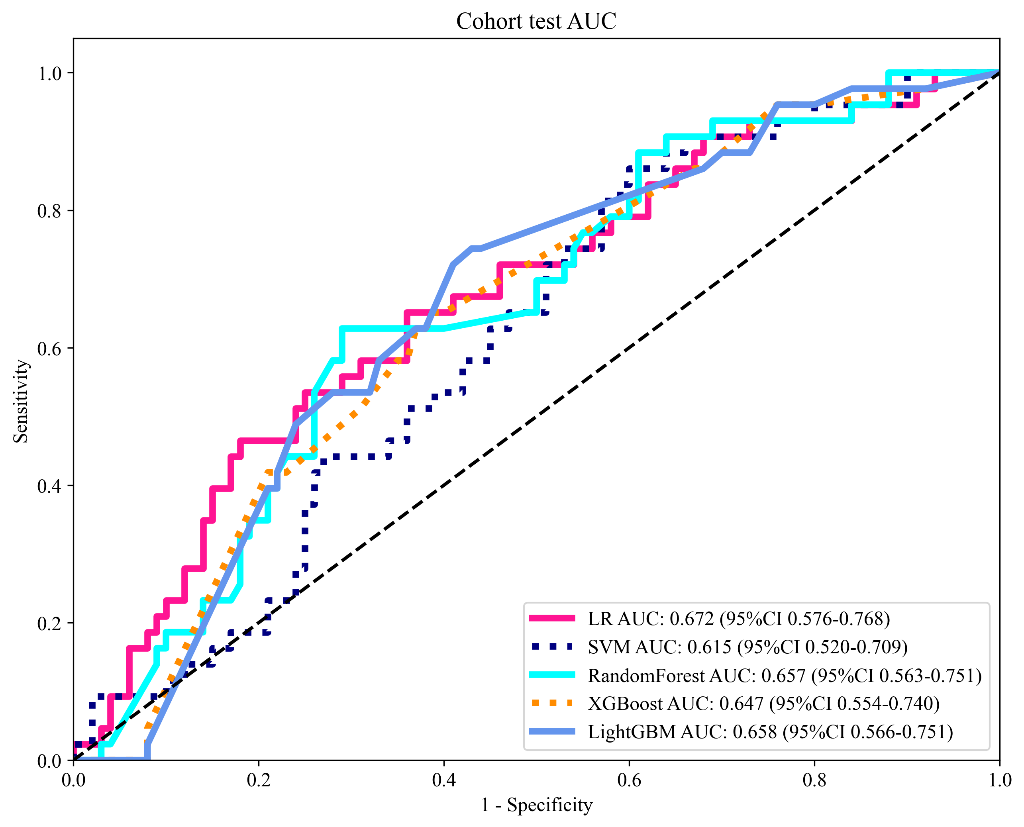

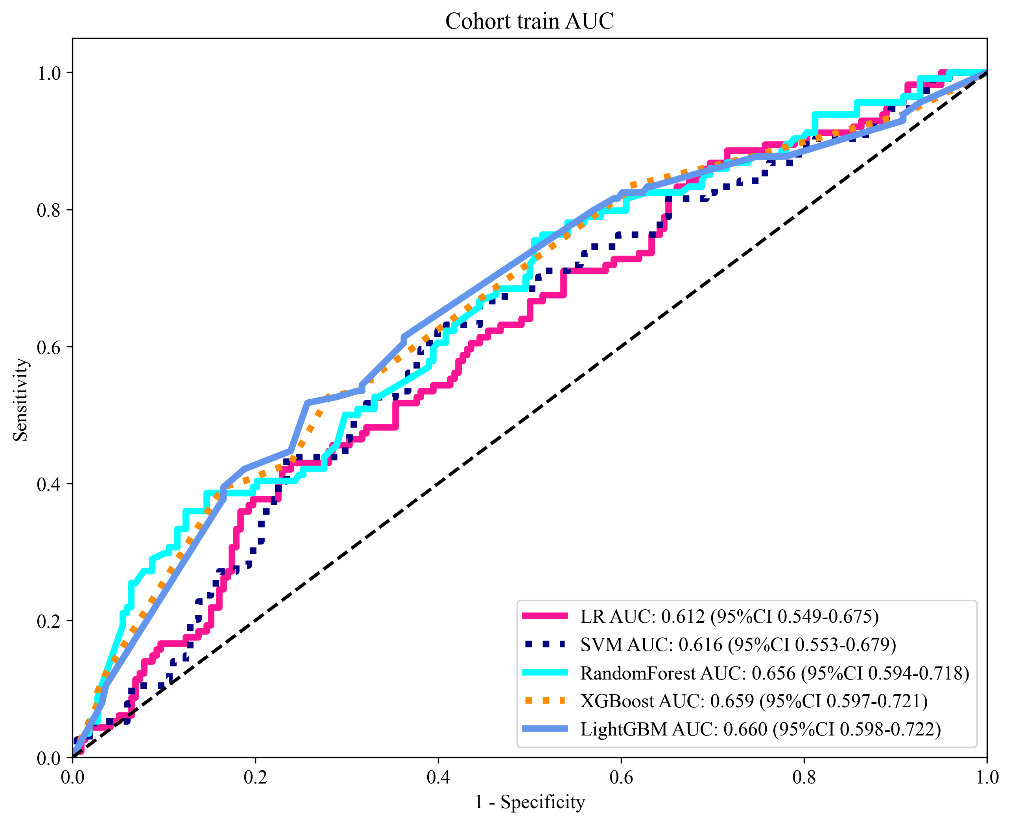
Supplementary Figure S4**.

Receiver operating characteristic (ROC) curves comparing radiomics and habitat models using the LightGBM algorithm.

(A) Training cohort.

(B) Validation cohort.


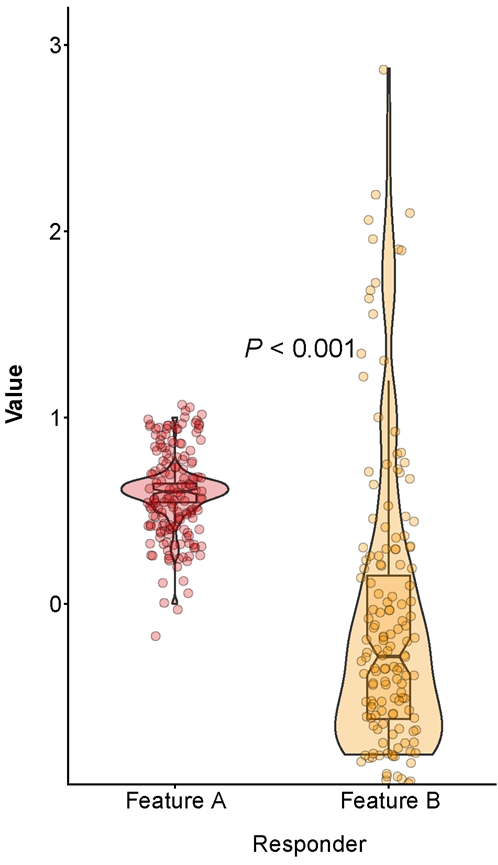

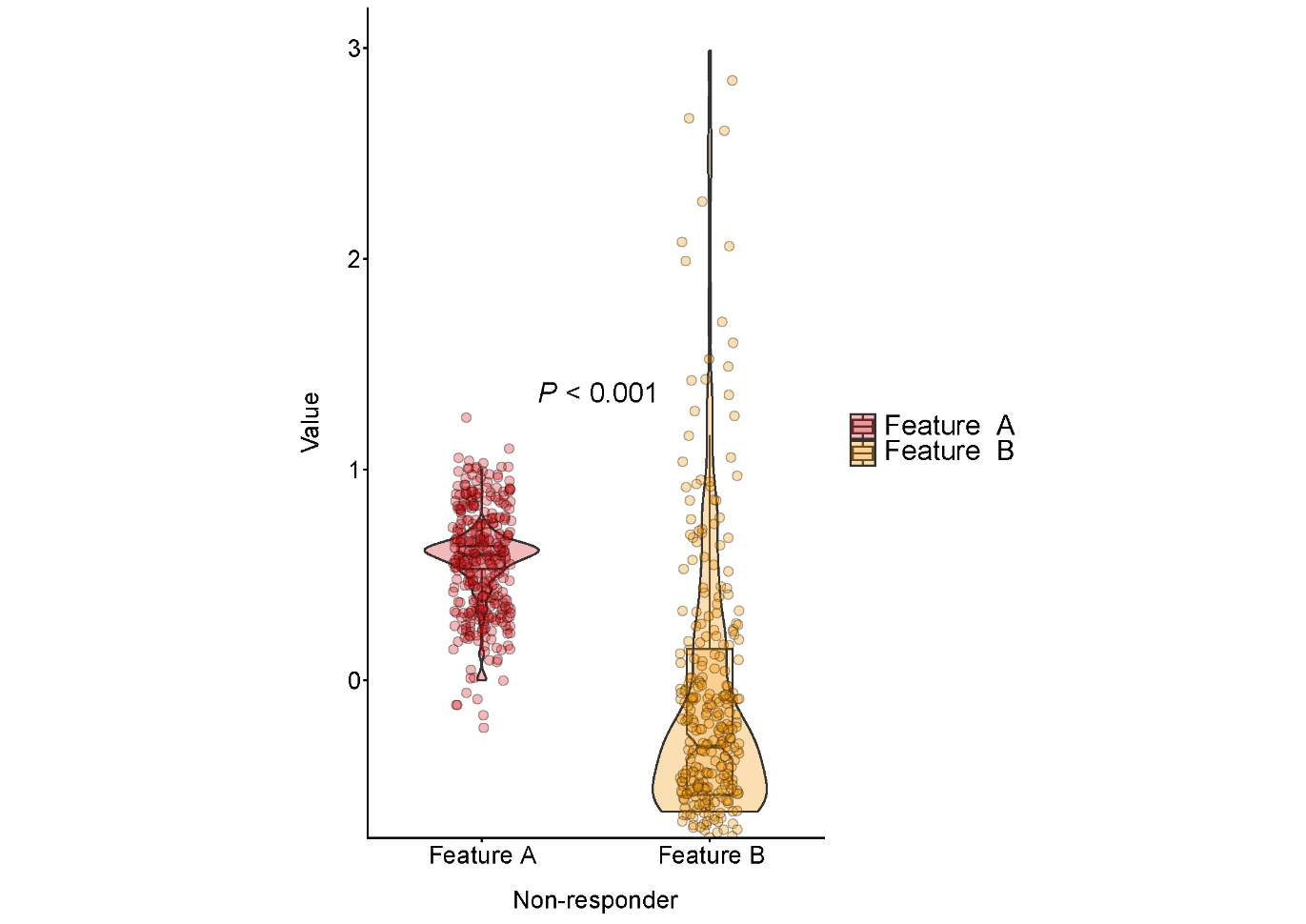


**Supplementary Figure S5.**

Distribution of key habitat features between responders and non-responders.

(A) wavelet_LHH_glszm_SmallAreaEmphasis_h3.

(B) wavelet_LLL_glszm_ZoneVariance_h3.


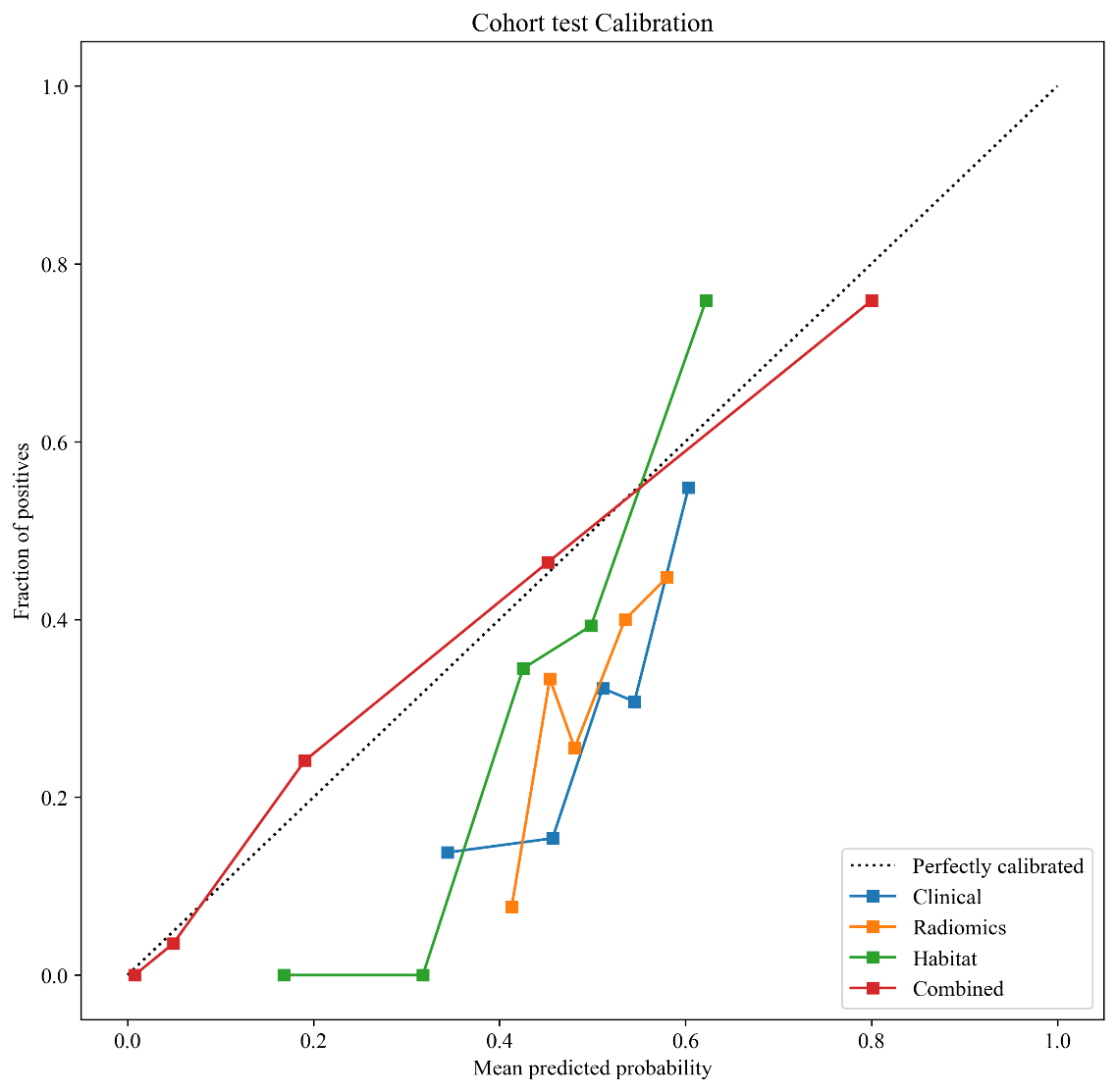
**
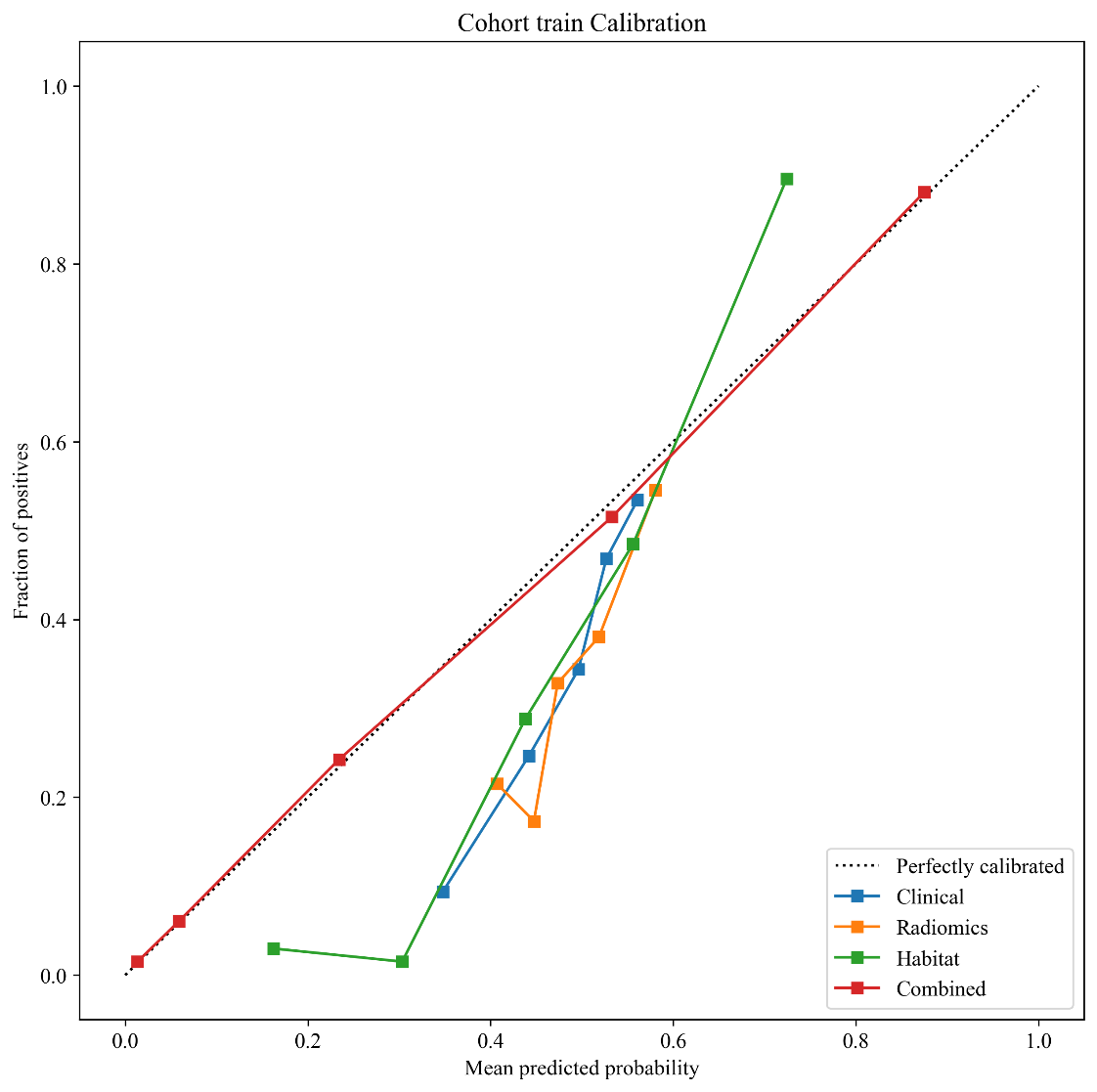
** **Supplementary Figure S6.**

Calibration curves of all models. (A) Training cohort. (B) Validation cohort.


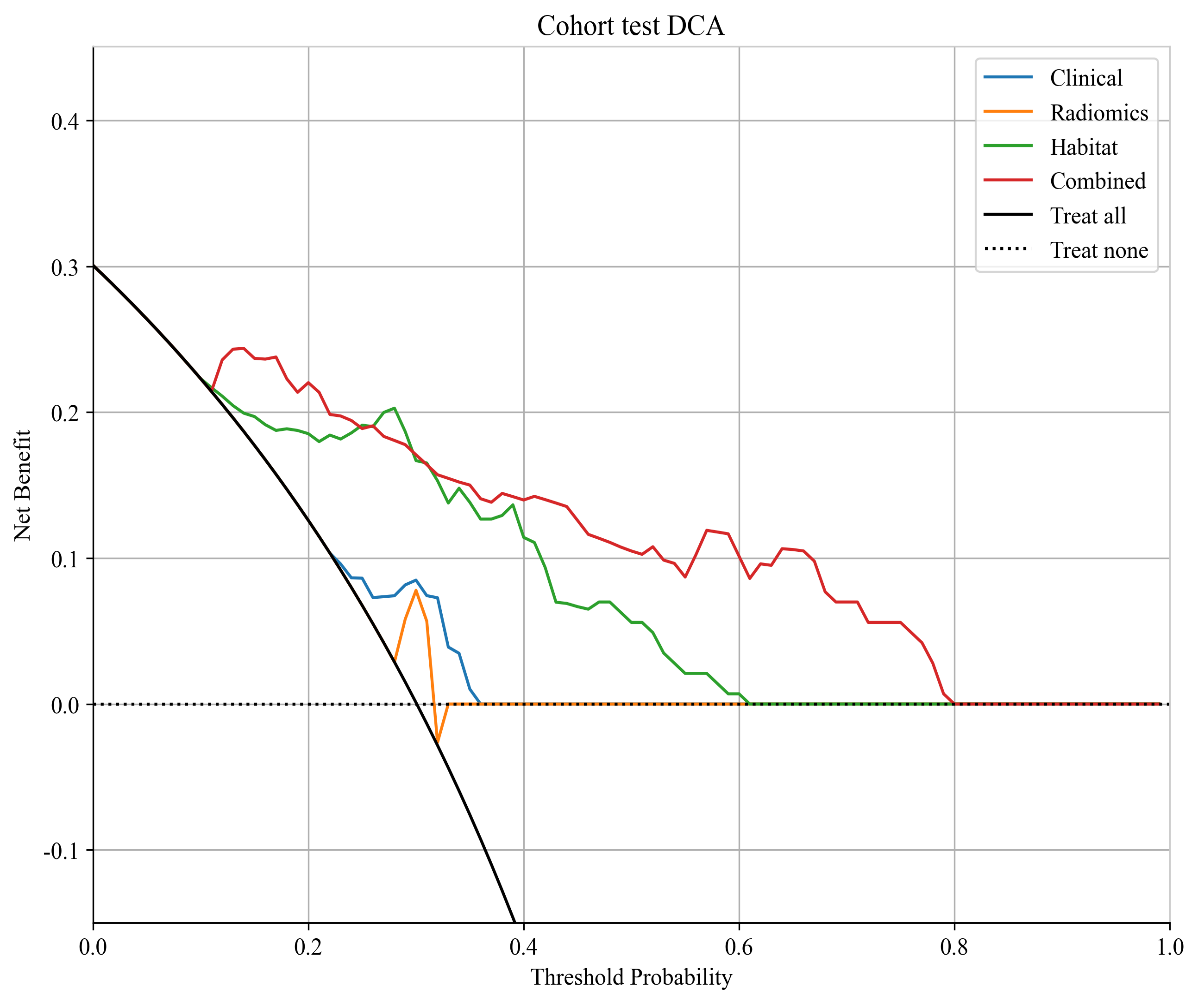

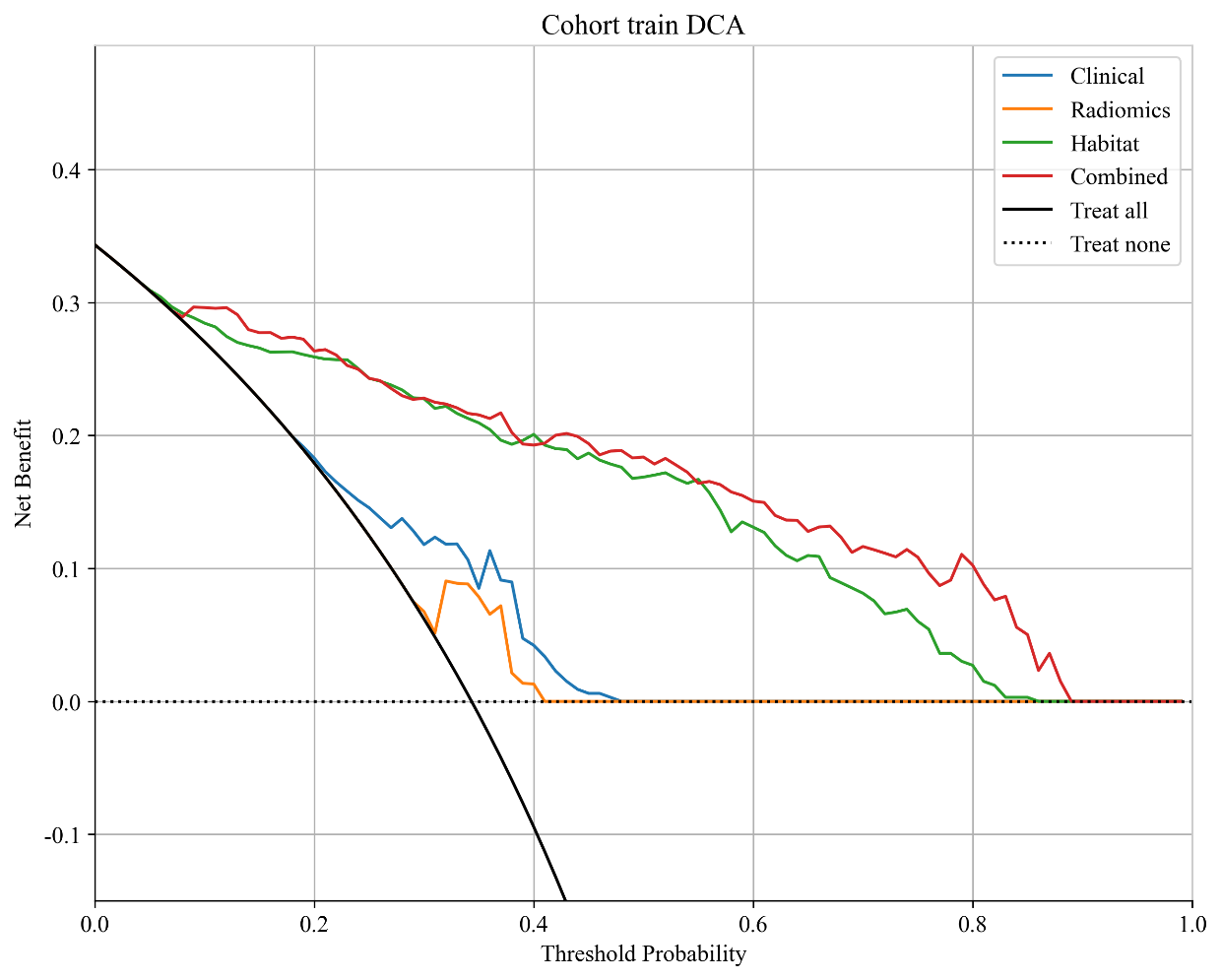
**Supplementary Figure S7.**

Decision curve analysis (DCA) of all models.

(A) Training cohort.

(B) Validation cohort.


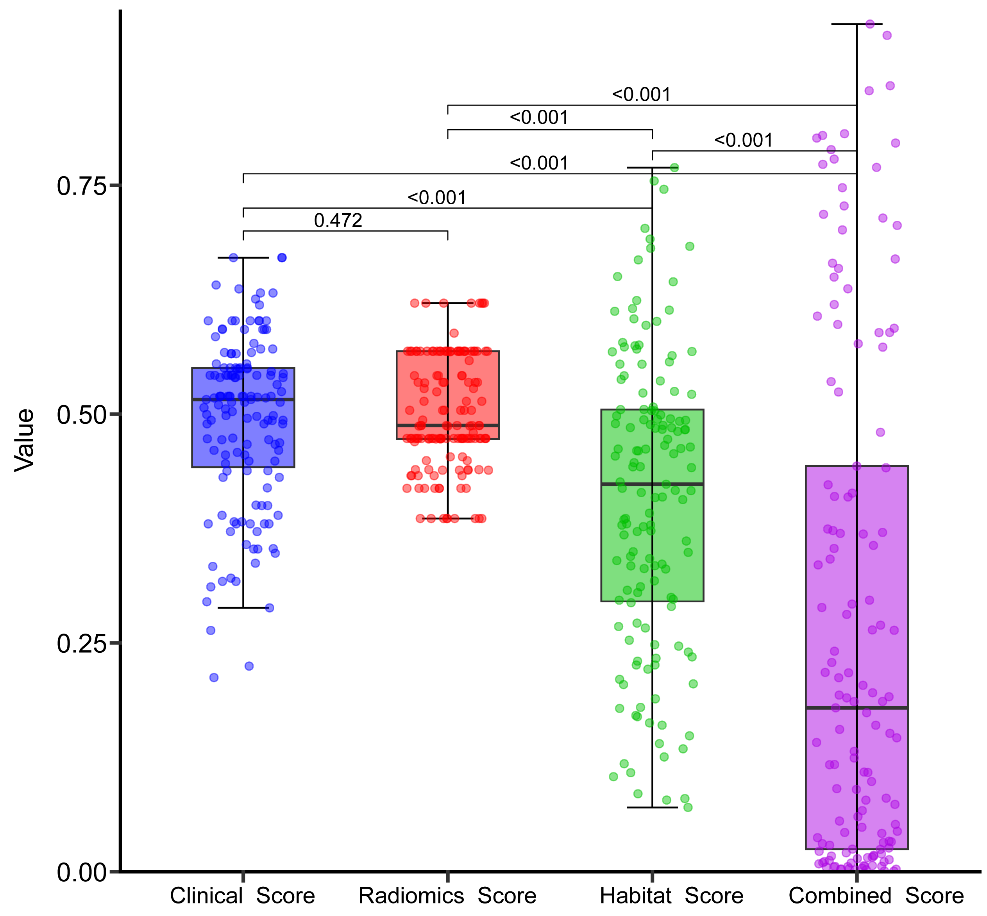

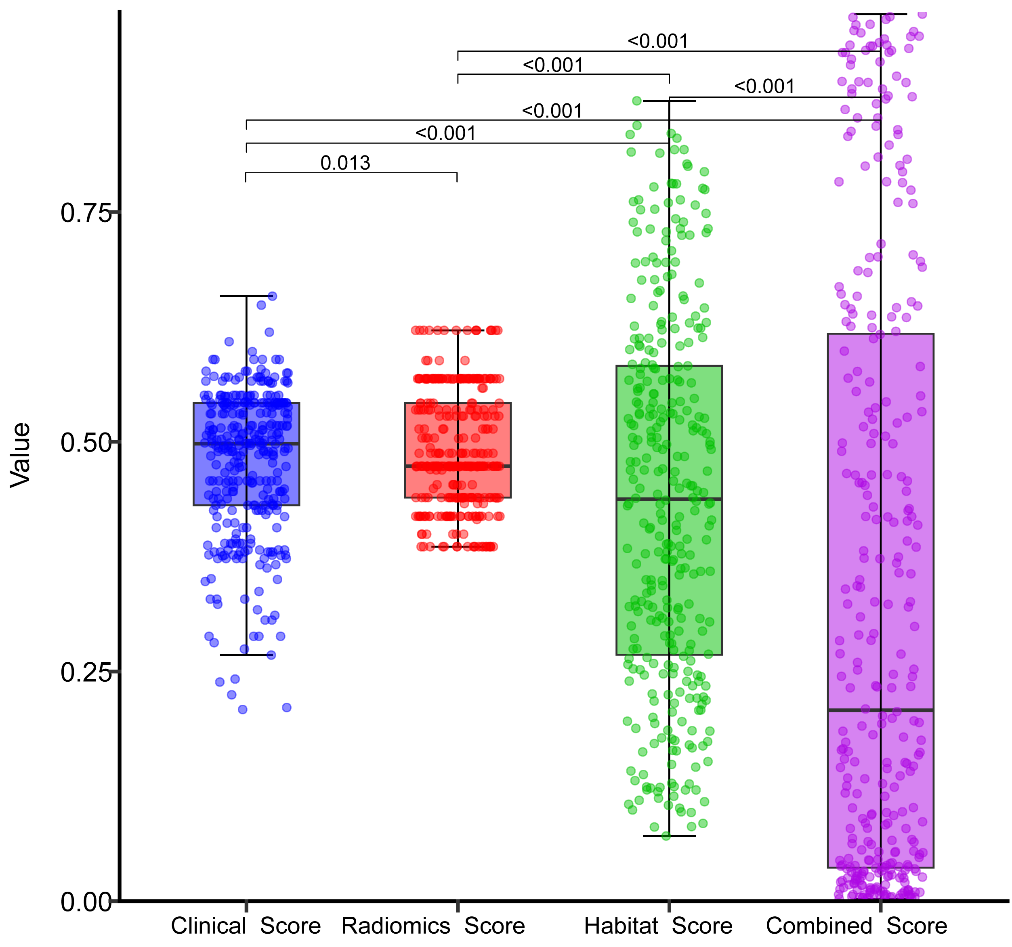


**Supplementary Figure S8.**

Distribution of predictive scores for different models.

(A) Training cohort.

(B) Validation cohort.

**
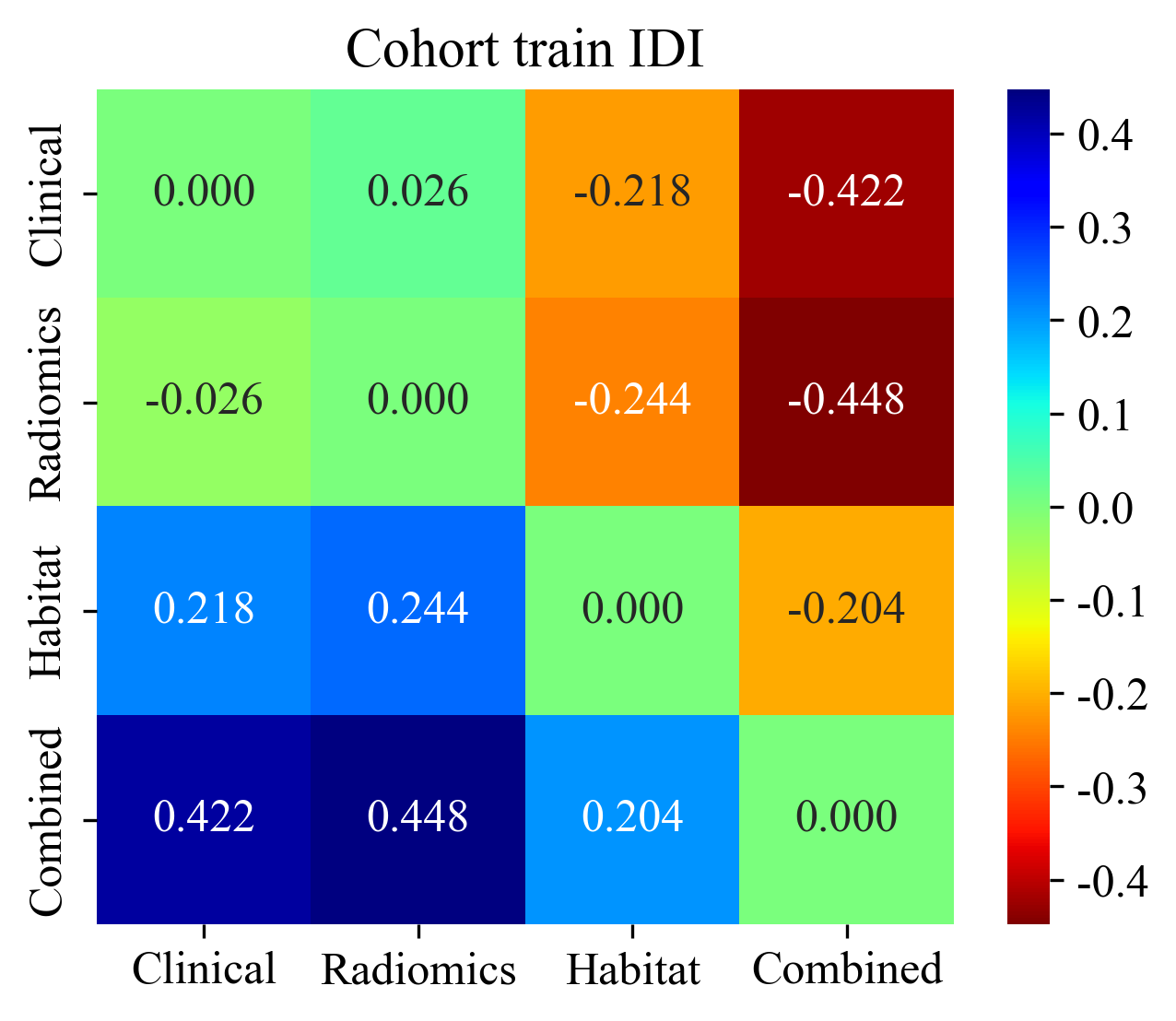

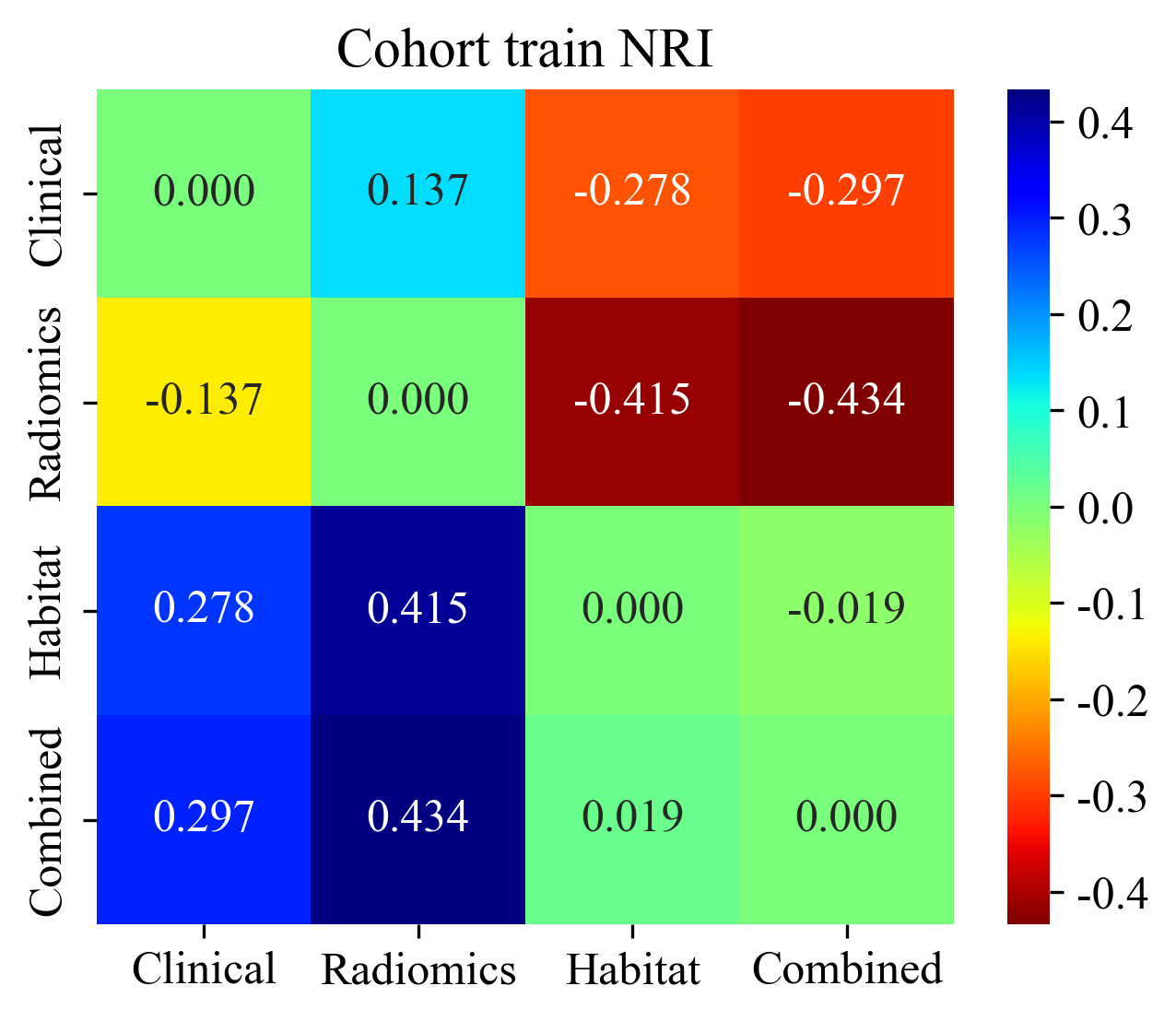
Supplementary Figure S9**.

Pairwise model comparison using heatmaps in the training cohort.

(A) Net Reclassification Improvement (NRI).

(B) Integrated Discrimination Improvement (IDI).

**Supplementary Figure S
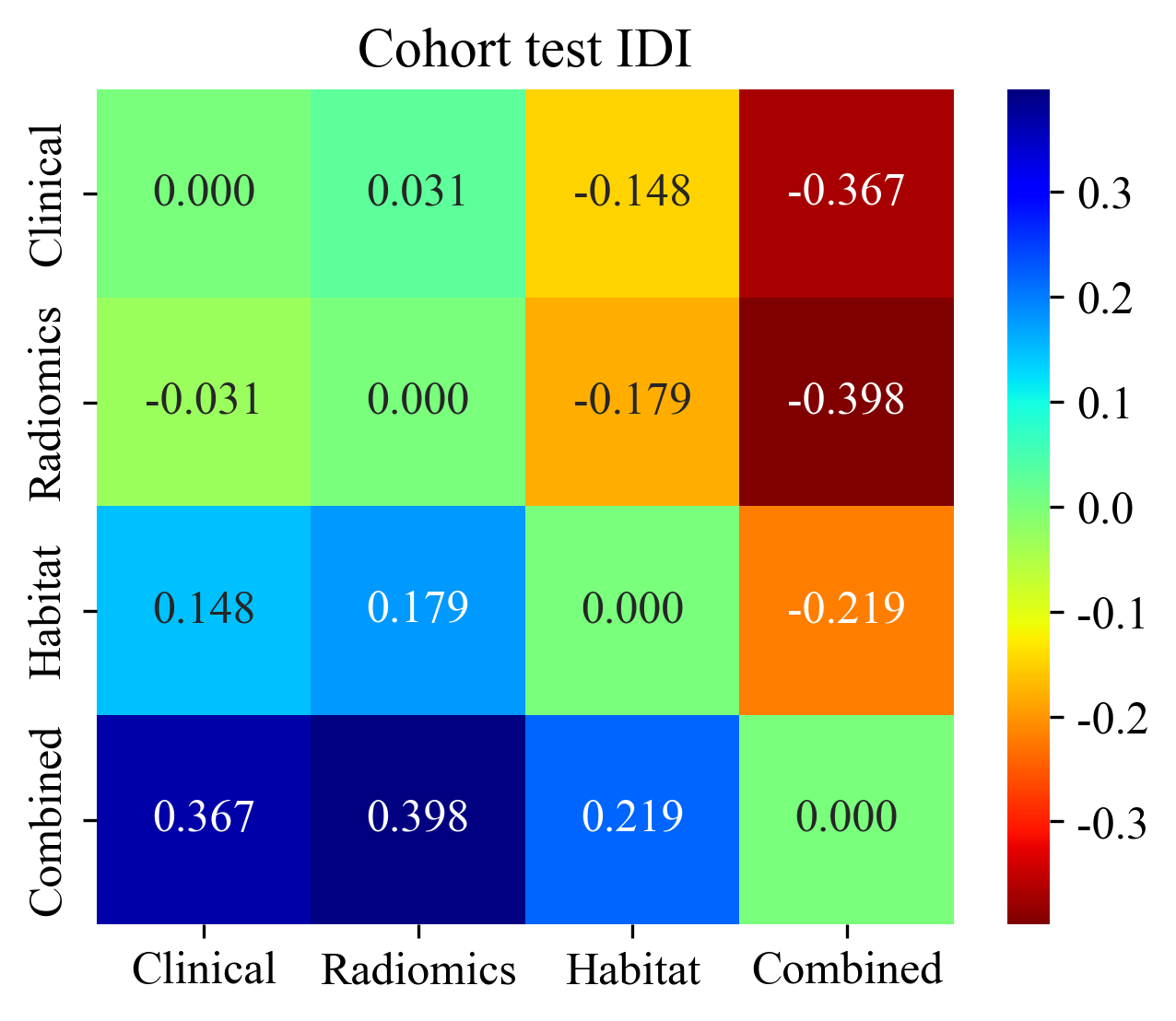

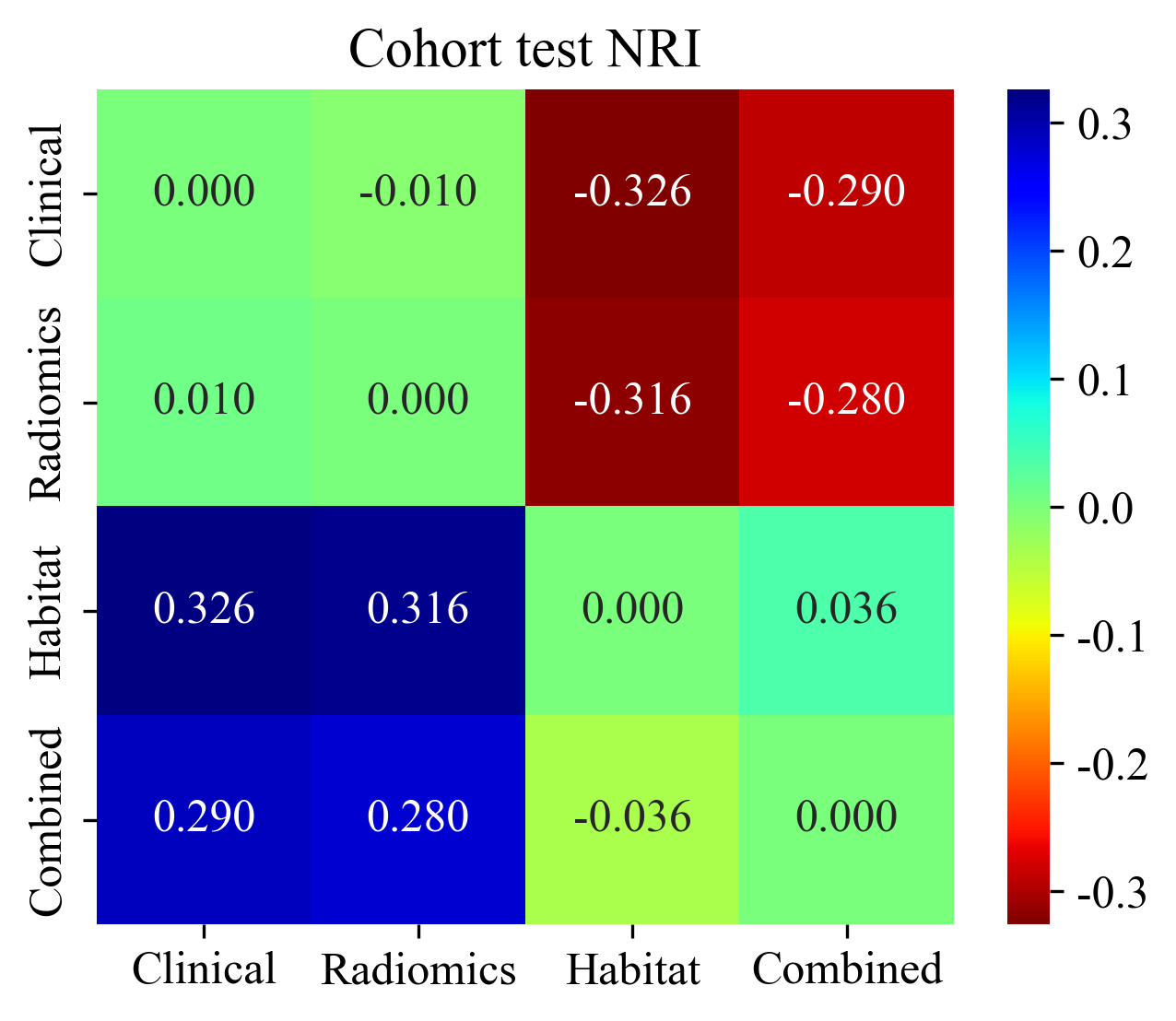
10.**

Pairwise model comparison using heatmaps in the validation cohort.

(A) Net Reclassification Improvement (NRI).
(B) Integrated Discrimination Improvement (IDI).

**
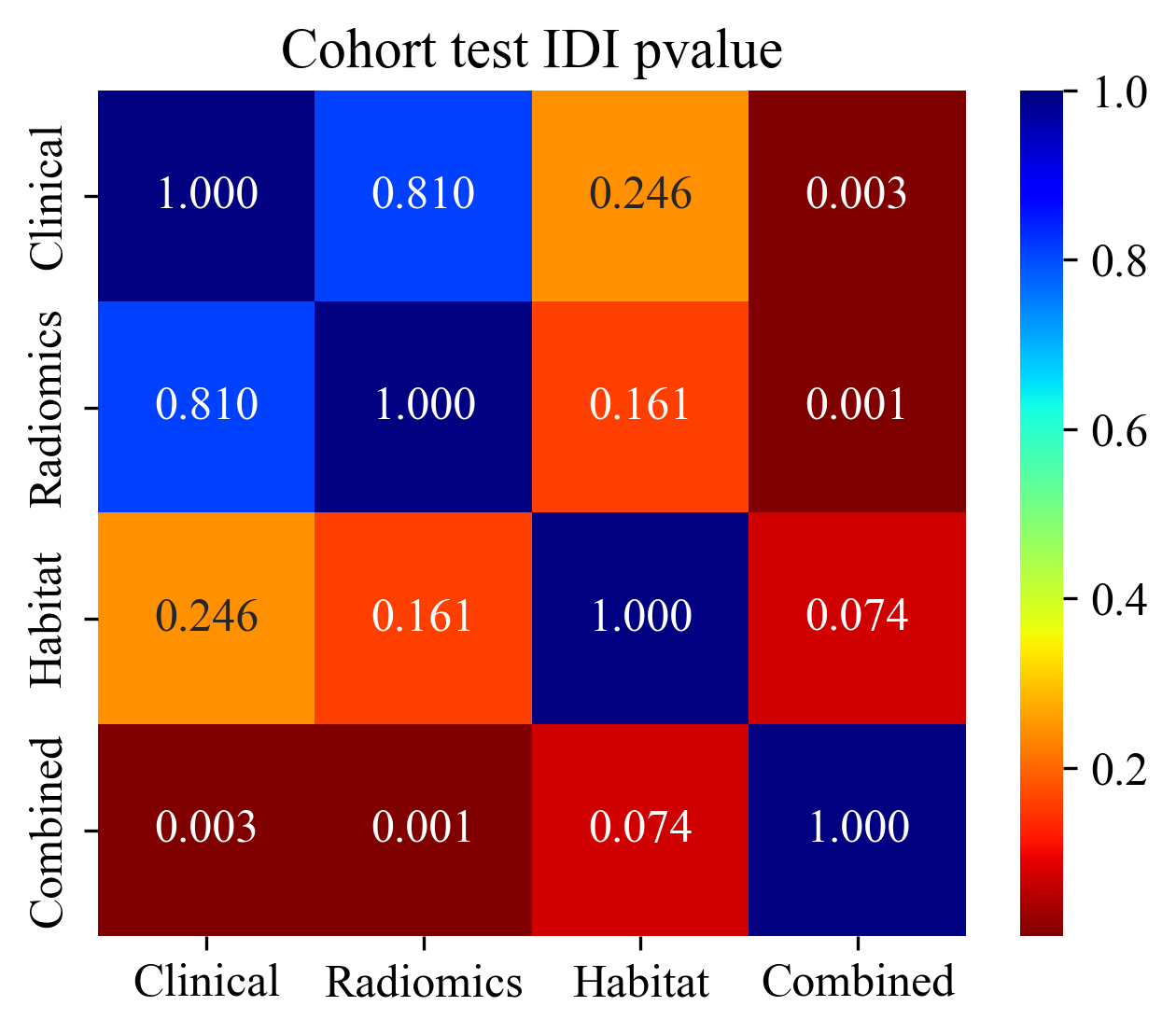

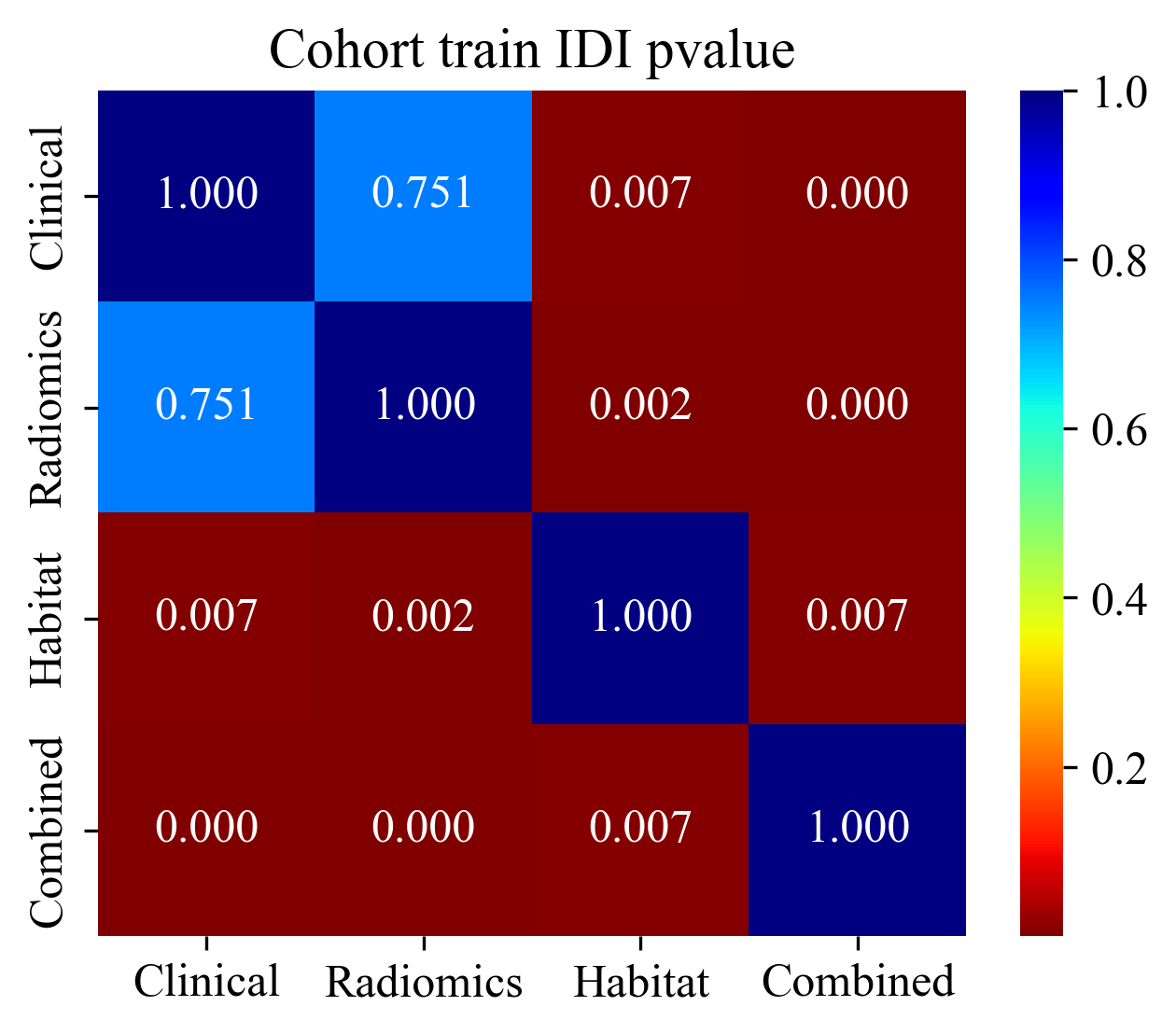
Supplementary Figure S11**.

Statistical significance of Integrated Discrimination Improvement (IDI) across models.
(A) Training cohort.
(B) Validation cohort.
